# Acute Activation of Metabolic Syndrome Components in Pediatric Acute Lymphoblastic Leukemia Patients Treated with Dexamethasone

**DOI:** 10.1371/journal.pone.0158225

**Published:** 2016-06-30

**Authors:** Lidewij T. Warris, Erica L. T. van den Akker, Marc B. Bierings, Cor van den Bos, Christian M. Zwaan, Sebastiaan D. T. Sassen, Wim J. E. Tissing, Margreet A. Veening, Rob Pieters, Marry M. van den Heuvel-Eibrink

**Affiliations:** 1 Department of Pediatric Oncology, Erasmus MC- Sophia Children’s Hospital, Rotterdam, The Netherlands; 2 Department of Pediatric Endocrinology, Erasmus MC- Sophia Children’s Hospital, Rotterdam, The Netherlands; 3 Department of Pediatric Oncology, Academic Medical Center – Emma Children’s Hospital, Amsterdam, The Netherlands; 4 Department of Pediatric Hematology and Oncology, VU Medical Center, Amsterdam, The Netherlands; 5 Department of Pediatric Hematology and Oncology, University Medical Center Utrecht – Wilhelmina Children’s Hospital, Utrecht, The Netherlands; 6 Department of Pediatric Hematology and Oncology, University of Groningen Medical Center, Groningen, The Netherlands; 7 Princess Máxima Center for Pediatric Oncology, Utrecht, The Netherlands; German Cancer Research Center (DKFZ), GERMANY

## Abstract

Although dexamethasone is highly effective in the treatment of pediatric acute lymphoblastic leukemia (ALL), it can cause serious metabolic side effects. Because studies regarding the effects of dexamethasone are limited by their small scale, we prospectively studied the direct effects of treating pediatric ALL with dexamethasone administration with respect to activation of components of metabolic syndrome (MetS); in addition, we investigated whether these side effects were correlated with the level of dexamethasone. Fifty pediatric patients (3–16 years of age) with ALL were studied during a 5-day dexamethasone course during the maintenance phase of the Dutch Childhood Oncology Group ALL-10 and ALL-11 protocols. Fasting insulin, glucose, total cholesterol, HDL, LDL, and triglycerides levels were measured at baseline (before the start of dexamethasone; T1) and on the fifth day of treatment (T2). Dexamethasone trough levels were measured at T2. We found that dexamethasone treatment significantly increased the following fasting serum levels (*P*<0.05): HDL, LDL, total cholesterol, triglycerides, glucose, and insulin. In addition, dexamethasone increased insulin resistance (HOMA-IR>3.4) from 8% to 85% *(P*<0.01). Dexamethasone treatment also significantly increased the diastolic and systolic blood pressure. Lastly, dexamethasone trough levels (*N* = 24) were directly correlated with high glucose levels at T2, but not with other parameters. These results indicate that dexamethasone treatment acutely induces three components of the MetS. Together with the weight gain typically associated with dexamethasone treatment, these factors may contribute to the higher prevalence of MetS and cardiovascular risk among survivors of childhood leukemia who received dexamethasone treatment.

## Introduction

Glucocorticoids are an important for effectively treating pediatric acute lymphoblastic leukemia (ALL). [1] Unfortunately, however, the high doses of dexamethasone typically used in ALL treatment protocols often give rise to severe side effects [2, 3], and acute metabolic side effects are more common in dexamethasone-based treatment protocol compared to prednisone-based protocols. [4, 5]

Metabolic side effects such as weight gain, altered fat distribution, hypertension, hyperglycemia, dyslipidemia, and altered insulin resistance occur in 10–45% of children treated with glucocorticoids. [2, 6–8] These effects can be enhanced by several factors, including increased energy intake due to food obsession, and reduced physical activity. [3, 9] In the long term, these metabolic side effects may contribute to a higher risk of developing metabolic syndrome (MetS) among survivors. [10–13] A recent study of 784 ALL survivors showed that 34% developed MetS, as defined by the National Cholesterol Education Program—Adult Treatment Panel III criteria. [14]

Although prednisolone affects metabolism, dexamethasone has a more severe toxicity profile [2, 15] acutely causing significant metabolic changes, including increased energy intake and insulin resistance. For example, a recent study reported the development of insulin resistance in 18 pediatric ALL patients after only five days of dexamethasone treatment. [6] Thus, short-term dexamethasone treatment can affect several components of the MetS, which is defined for children ≥10 years of age as abdominal obesity plus at least two of the following clinical features: hyperglycemia, hypertriglyceridemia, decreased HDL cholesterol, and hypertension. [16] Esbenshade *et al*. longitudinally followed 34 pediatric ALL patients for one year of maintenance therapy containing prednisone or dexamethasone and documented increased weight gain, insulin resistance, and leptin levels during the course of the year. [17] These striking findings underscore the long-term consequences of acute glucocorticoid-induced metabolic toxicity. Thus, given that the Dutch Childhood Oncology Group (DCOG) ALL protocols call for dexamethasone pulses to be administered for one and a half years during the maintenance phase, it is conceivable that the risk factors for developing MetS accumulate during the course of treatment. However, prospective studies regarding the direct, acute effects of dexamethasone on MetS components in a substantial cohort of pediatric ALL patients have not been performed. Moreover, no information is currently available regarding the role of dexamethasone pharmacokinetics in the occurrence of MetS. To address these questions, we prospectively measured the acute effects of dexamethasone on all components of MetS in the context of a randomized controlled trial, and we examined the correlation between serum dexamethasone levels and these components in a subset of patients.

## Materials and Methods

### Patients

The study protocol was approved by the Ethics Committee of Erasmus Medical Center and the local ethics committees at the following participating centers (MEC-2012-155/ EudraCT 2011-003815-46): University Medical Center Utrecht, Academic Medical Center Amsterdam, VU Medical Center, and University of Groningen Medical Center. In accordance with national regulations, the parents and/or legal guardians of patients provided written informed consent; patients 12–16 years of age also provided their written informed consent. Fifty pediatric patients 3–16 years of age with ALL who were enrolled in the Dexadays study (NTR3280, a multicenter, randomized controlled trial; S1 File. Study Protocol), were included in the present study. [18]

**Fig 1:**
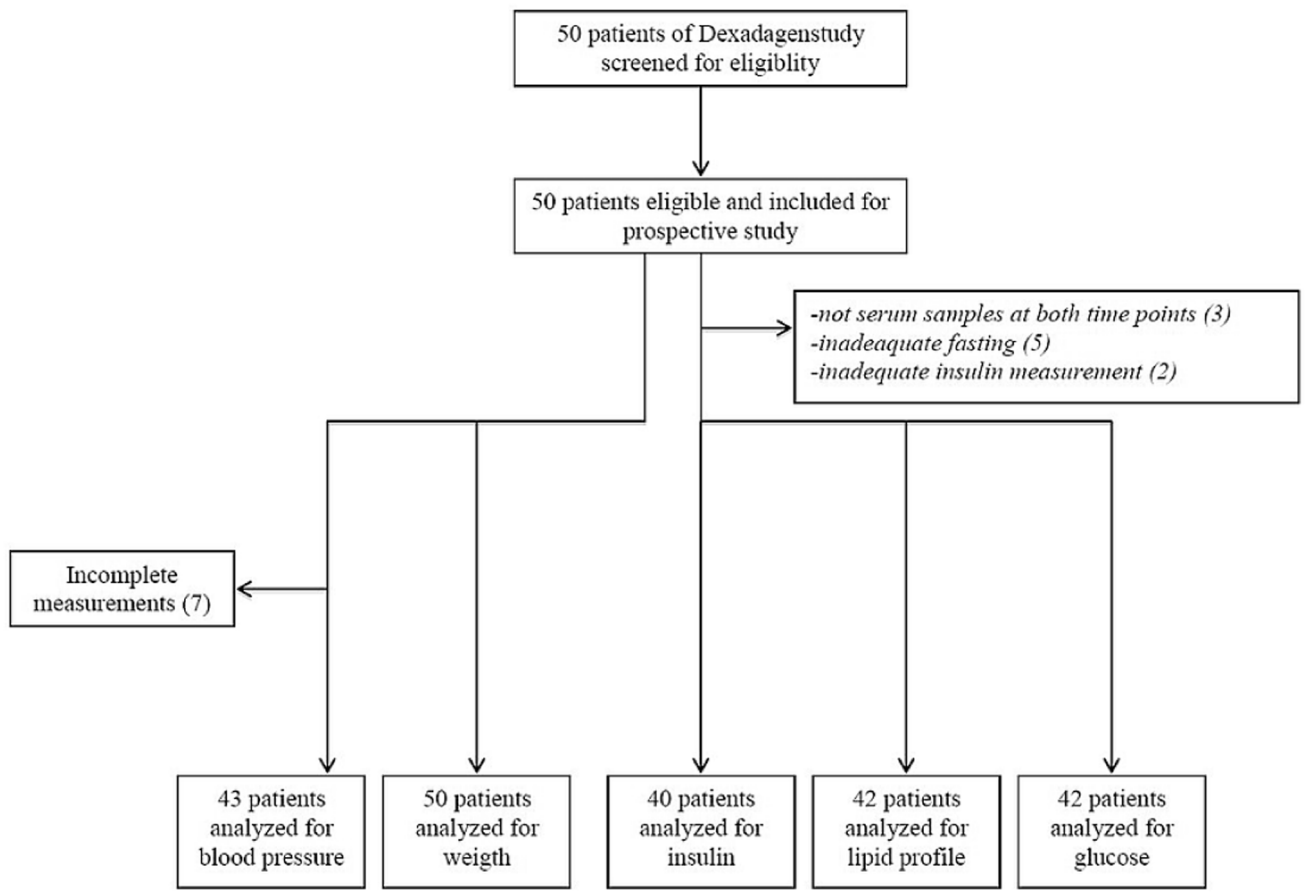
Consort flowchart.

Patients received dexamethasone pulses during the maintenance phase of treatment in accordance with DCOG ALL protocols at five pediatric oncology departments in the Netherlands. In the present study, patients were included only after the asparaginase phase was discontinued, as asparaginase treatment can cause hypoalbuminemia, is associated with increased plasma exposure to dexamethasone [19], can influence glucose metabolism, and can cause changes in lipid levels. In the part of the randomized crossover trial (NTR3280) in which we performed the present study (Fig 1), patients underwent a 5-day course of oral dexamethasone (6 mg/m^2^/day) with the addition of a placebo (10 mg/m^2^/day)(containing 0.26 g/mL sorbitol 70% solution). [18] Dexamethasone was given together with 2 mg/m^2^ vincristine on day 1, 50 mg/m^2^ 6-mercaptopurine daily, and 30 mg/m^2^ methotrexate weekly. Our primary outcome was metabolic function measured after four days of dexamethasone treatment.

### Anthropometry

Baseline height (in meters), weight (standard deviation score (SDS)), and waist circumference (in cm) were measured before the start of the dexamethasone course (T1). Blood pressure (in mmHg) was measured at T1 and on the morning of the fifth day of dexamethasone treatment (T2). Blood pressure was measured in the right arm with the subject in the sitting position after one hour at rest (Dinamap^®^ Procare). Cut-off values for these anthropometric parameters corrected for age and sex were obtained from Cole *et al*. [20] (for BMI: adiposity, obesity), the reference values of the National High Blood Pressure Education Program Working Group [21] (for hypertension), and Fredriks *et al*. [22] (for waist circumference: >1.3 SD = abdominal adiposity, >2.3 SD = abdominal obesity). Blood pressure was expressed as SDS adjusted for height and sex. [23] Hypertension was defined as either systolic or diastolic blood pressure above the 95^th^ percentile for the patient’s age and sex. [21]

### Laboratory measurements

At T1 and T2, peripheral blood samples were obtained following an overnight fast. These samples were then used to measure serum insulin, glucose, total cholesterol, high-density lipoprotein (HDL) cholesterol, low-density lipoprotein (LDL) cholesterol, and triglyceride levels. Laboratory reference values from the Department of Clinical Chemistry at Erasmus Medical Center [24] and the International Diabetes Federation [16] were used. The serum samples were stored at -80°C and were analyzed together at the end of the study. Serum samples were measured using an enzymatic *in vitro* assay (Roche Diagnostics, Mannheim, Germany). [25]

Impaired fasting glucose (IFG) was defined as a fasting glucose value ≥5.6 mmol/L and <7.0 mmol/L in accordance with the criteria established by the America Diabetes Association. [26] Hyperinsulinemia was defined as a fasting insulin value ≥15 mU/L (for prepubescent children), ≥30 mU/L (for pubescent children), or ≥20 mU/L (for post-pubescent children). [27] The insulin resistance index was calculated using the homeostasis model assessment of insulin resistance (HOMA-IR) method, which is calculated using the following formula: [fasting glucose (mmol/L)*fasting insulin (mU/L)/22.5]. [28] A cut-off value of HOMA-IR ≥ 3.4 was chosen because of its predictive value for impaired glucose tolerance and diabetes mellitus. [29–34] We also repeated out analysis using a cut-off value of >4.39 for insulin resistance in order to compare our results with the results reported by Chow *et al*. [6]

Metabolic syndrome (MetS) was defined by the International Diabetes Federation (IDF) criteria for children ≥10 years of age with abdominal obesity (waist circumference ≥90 percentile), plus at least two of the clinical features: hyperglycemia, hypertriglyceridemia, reduced HDL cholesterol, hypertension. [16] Due to these age restrictions we could not use the IDF criteria to diagnose our patients with MetS, but we used the criteria to study the components that play a role in the development of MetS.

### Dexamethasone pharmacokinetics

At T2, dexamethasone trough levels were measured at the Pharmacology department, at Academic Medical Center, Amsterdam. Dexamethasone was measured in the serum samples obtained on the morning of the fifth day of treatment, before dexamethasone treatment was administered (i.e., after four consecutive days of dexamethasone). The precise time of the oral dexamethasone treatment on the previous night was retrieved from the patient diaries.

### Statistics

Glucose and lipid levels were compared between T1 and T2 using the Student’s *t*-test, as these values were normally distributed. Insulin, HOMA-IR values, and blood pressure SDS values were compared between T1 and T2 using the Wilcoxon signed-rank test. Spearman’s coefficient (r) was used to determine the correlation between age and the following factors: weight SDS, BMI, glucose levels at T2, hyperinsulinemia at T1, change in diastolic blood pressure, hypertension (either diastolic or systolic) at T2, and systolic hypertension at T1. Spearman’s coefficient (r) was also used to determine the correlation between dexamethasone trough levels and glucose levels. All analyses were performed using SPSS, version 21 (IBM Corp., Armonk, NY).

## Results

### Baseline characteristics

A total of 50 pediatric patients (46% male) were included. (Fig 1) (S2 File. Consort Checklist) Serum samples for laboratory measurements were missing for three patients. The median age of the patient cohort was 6.0 years (interquartile range (IQR): 4.0, 10.3). Based on BMI, adiposity was present in 7% of patients at the time of diagnosis, and this increased to a prevalence of 19% at baseline (T1). Based on waist circumference >1.3 SD, 48% of the patients had abdominal adiposity at baseline (T1). Based on BMI, obesity was not present in any of the patients (0%) at the time of diagnosis, and this increased to a prevalence of 4% at baseline (T1). Based on waist circumference >2.3 SD, 10% of the patients had abdominal obesity at baseline. Both weight SDS (r = 0.44, *P*<0.01) and high BMI (r = 0.35, *P* = 0.02) were significantly correlated with age.

### Glucose metabolism

**Fig 2:**
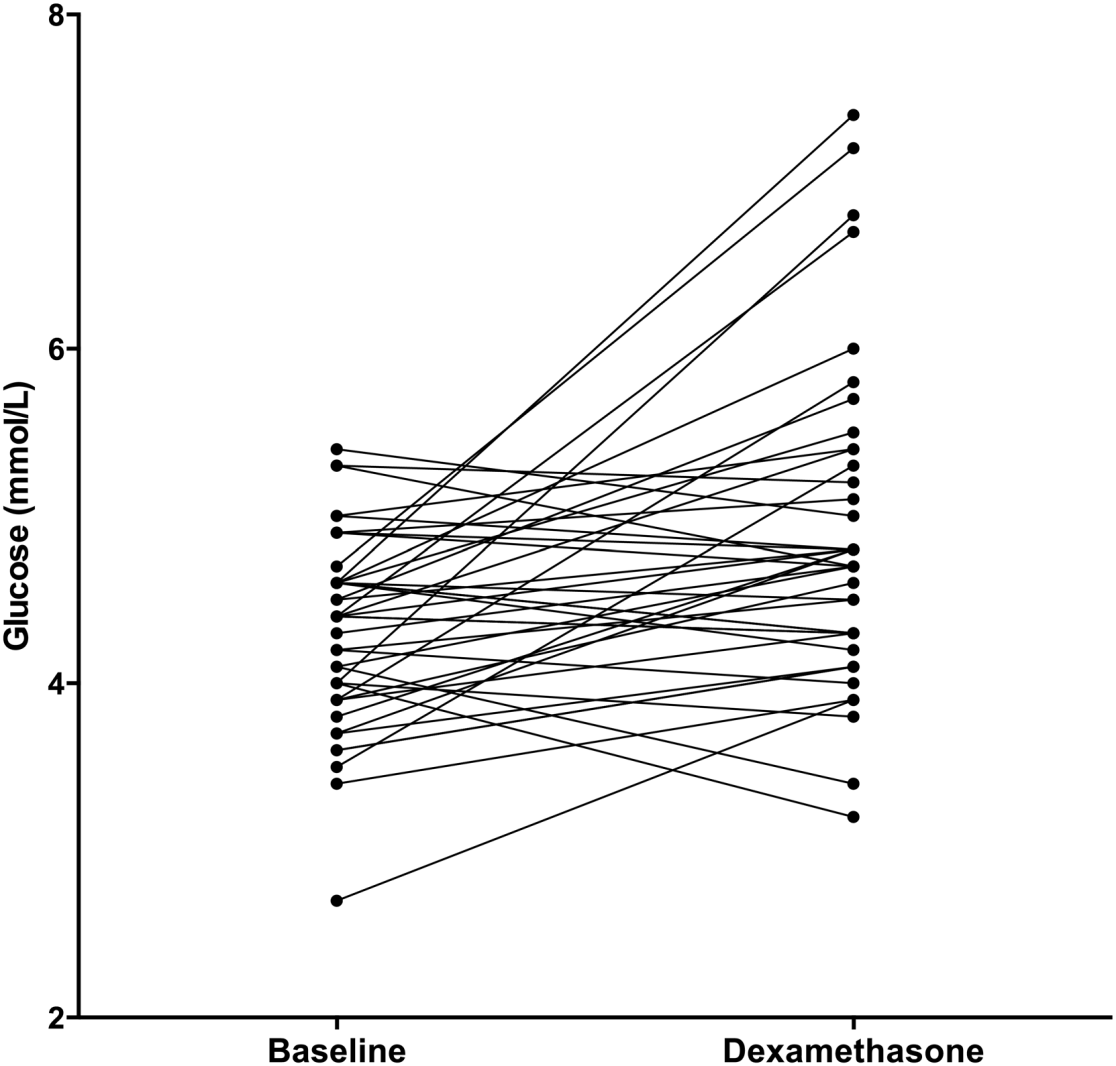
Glucose. Fasting glucose values at baseline (T1) and after four days of dexamethasone treatment (T2). N = 42.

The serum samples from five patients were excluded from our analysis due to insufficient fasting prior to collection. The fasting glucose levels (*N* = 42) increased significantly (Fig 2) after four days of dexamethasone treatment (median increase: 0.40 mmol/L (IQR: -0.20, 1.03), *P*<0.01). None of the patients had impaired fasting glucose (IFG) [28] at baseline; after four days of dexamethasone treatment, two patients (5%) had IFG (*P* = 0.32). (Table 1) Higher fasting glucose levels at T2 were significantly correlated with higher age (r = 0.49, *P* = 0.03). In contrast, we found no correlation between glucose levels and sex or week of maintenance phase.

**Table 1:** Laboratory measurements.

	Before Dexamethasone	After 4 days of Dexamethasone	P-value
**Glucose (mmol/L)** Median (IQR)	4.40 (3.90, 4.60)	4.70 (4.28, 5.33)	<0.01
**Hyperglycemia** N(%)	0 (0)	2 (4.8)	0.96
**HDL** Median (IQR)	1.42 (1.17, 1.77)	1.55 (1.27, 1.82)	<0.01
**Low HDL** N(%)	2 (4.8)	0 (0)	0.01
**High HDL** N(%)	5 (11.9)	11 (26.2)	
**LDL** Median (IQR)	2.55 (2.02, 3.07)	2.76 (2.24, 3.47)	<0.01
**High LDL** N(%)	7 (16.7)	11 (26.2)	<0.01
**Total Cholesterol** Median (IQR)	4.20 (3.70, 4.90)	4.60 (4.05, 5.43)	<0.01
**High Total Cholesterol** N(%)	4 (9.5)	11 (26.2)	0.01
**Triglycerides** Median (IQR)	0.86 (0.67, 1.25)	1.09 (0.93, 1.35)	0.04
**Hypertriglyceridemia** N(%)	10 (23.8)	17 (40.5)	0.09
**Insulin (pmol/L)** Median (IQR)	25.15 (14.40, 73.43)	216.50 (158.25, 406.50)	<0.01
**High Insulin** N(%)	4 (10.0)	36 (90.0)	<0.01
**HOMA-IR** Median (IQR)	0.70 (0.41, 2.13)	6.76 (4.21, 12.79)	<0.01
**Insulin Resistance HOMA-IR > 4.39** N(%)	2 (5.1)	28 (71.8)	<0.01
**Insulin Resistance HOMA-IR>3.4** N(%)	3 (7.7)	33 (84.6)	<0.01

Change (= delta) in the indicated laboratory values form T1 (baseline) to T2 (following four daily dexamethasone treatments). The changes in lipids and glucose values (N = 42) were analyzed using the Student’s Paired t-test. The changes in insulin (N = 40) and HOMA-IR (N = 39) were analyzed using the Wilcoxon signed-rank test.

**Fig 3:**
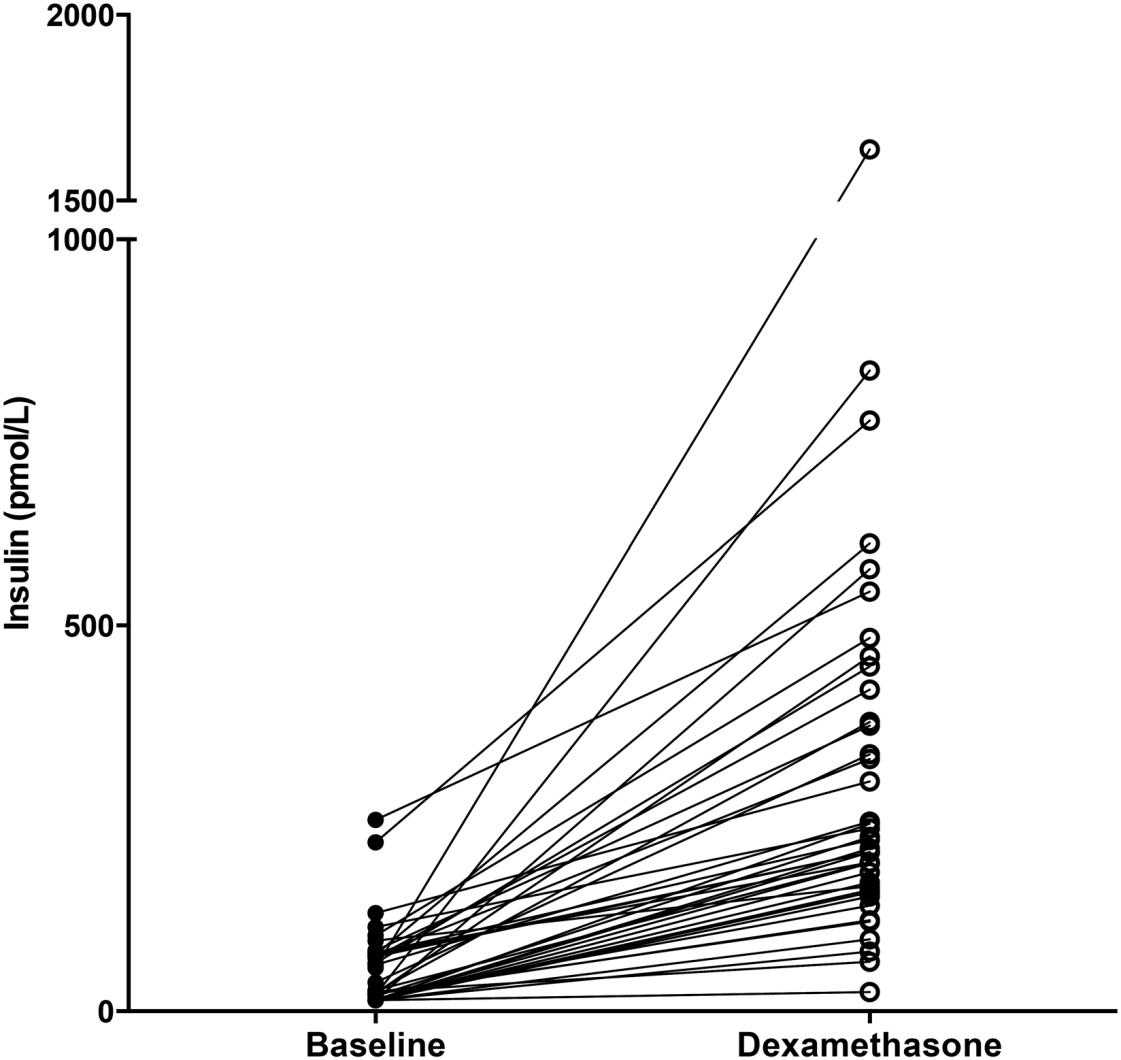
Insulin. Insulin values at baseline and after four days of dexamethasone treatment. N = 40.

**Fig 4:**
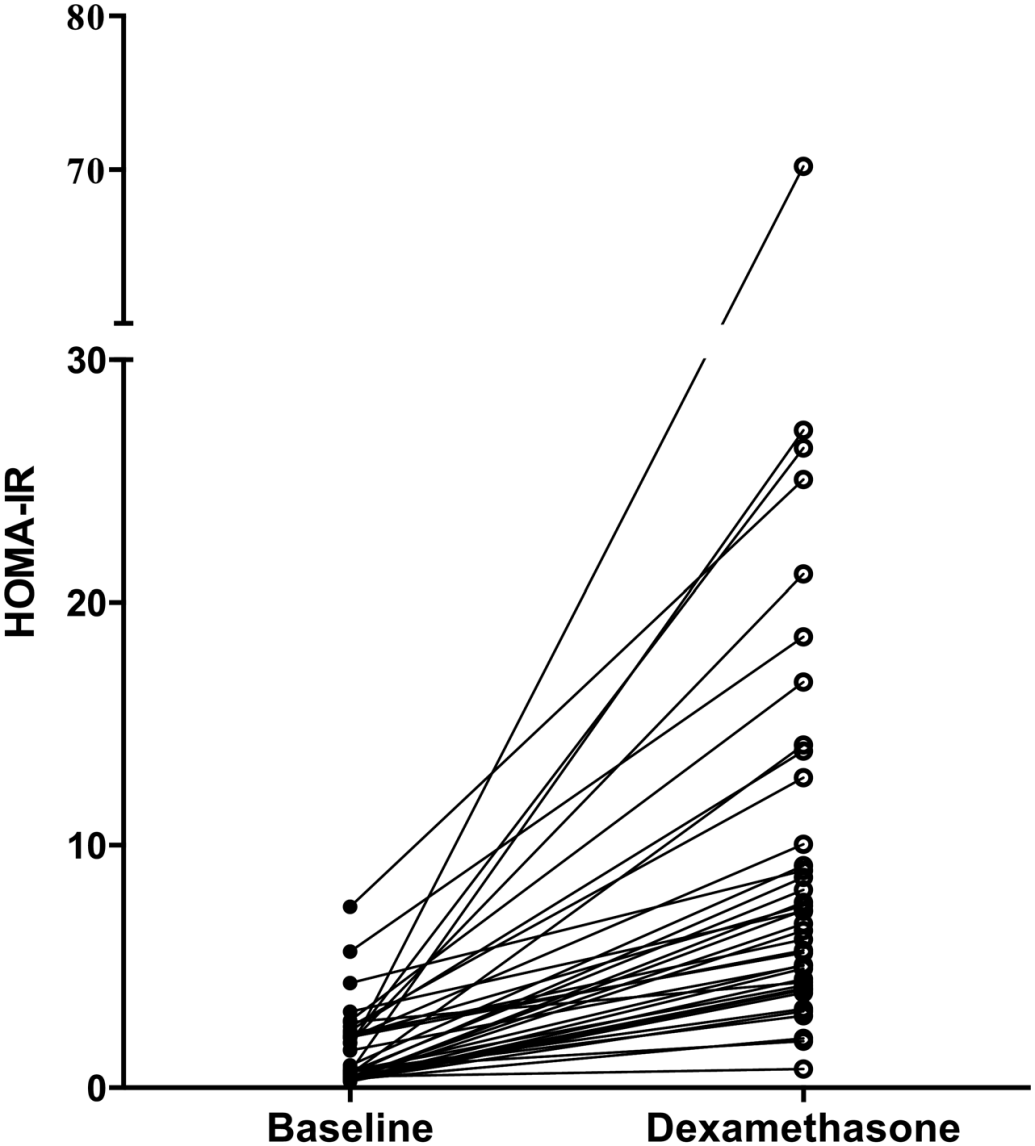
HOMA-IR. HOMA-IR values at baseline and after four days of dexamethasone treatment. N = 39.

The median insulin level at baseline (*N* = 40) was 25.2 pmol/L (IQR: 14.4, 73.4), and this value increased significantly to 173.75 pmol/L (IQR: 129.28, 334.55) after four days of dexamethasone treatment (*P*<0.01). (Fig 3) Hyperinsulinemia was present in 4 (10%) and in 36 (90%) patients at baseline and after 4 days of dexamethasone treatment, respectively (*P*<0.01). HOMA-IR values (*N* = 39) also increased significantly over the treatment course (median increase: 4.66 (IQR: 3.50, 10.27), *P*<0.01) (Fig 4). The prevalence of insulin resistance (defined as HOMA-IR >3.4) significantly increased from T1 (8%) to T2 (85%; *P*<0.01). When we repeated this analysis using the cut-off value used by Chow *et al*. (HOMA-IR > 4.39) [6], the increase in insulin resistance (from 5% to 72%) remained significant (*P*<0.01). We also found a significant correlation between hyperinsulinemia at baseline and age (r = 0.41, *P* = 0.01). None of the patients received insulin therapy during the dexamethasone course. Neither sex nor the week of maintenance phase was correlated with insulin resistance.

### Lipid profile

**Fig 5:**
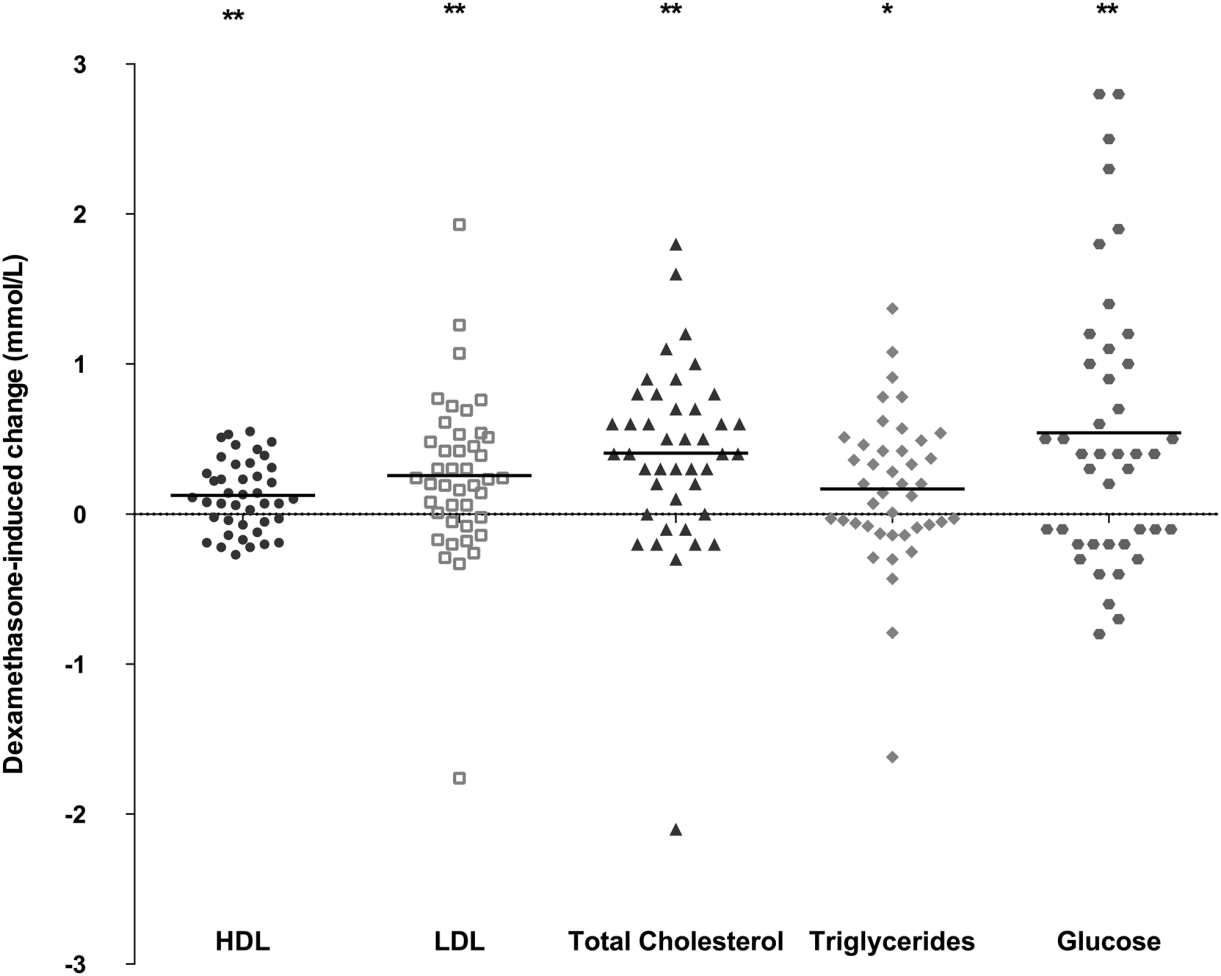
Lipids and glucose. Dexamethasone-induced change in HDL, LDL, total cholesterol, triglycerides, and glucose (mmol/L) from T1 to T2. * = P<0.05 and ** = P<0.01 (Student’s paired t-test). The horizontal lines indicate the median change.

The serum samples from five patients were excluded from the analysis due to insufficient fasting. Dexamethasone treatment increased the following fasting blood levels (*N* = 42): HDL (median increase: 0.11 mmol/L, IQR: -0.06, 0.32; *P*<0.01), LDL (median increase: 0.24 mmol/L, IQR: -0.03, 0.52; *P*<0.01), total cholesterol (median increase: 0.40 mmol/L, IQR: 0.08, 0.73; *P*<0.01), and triglycerides (median increase: 0.17 mmol/L, IQR: -0.08, 0.47; *P* = 0.04); these results are summarized in Fig 5 and Table 1. At baseline, 12%, 17%, 10%, and 24% of patients had high HDL, high LDL, high total cholesterol, and hypertriglyceridemia, respectively, and these percentages increased to 26%, 26%, 26%, and 41%, respectively, after four days of dexamethasone treatment (Table 1). Dexamethasone administration did not result in a decrease in HDL, which is usually seen in MetS. Lastly, serum lipid levels were not correlated with sex, age, or the week of maintenance phase.

### Blood pressure

**Fig 6:**
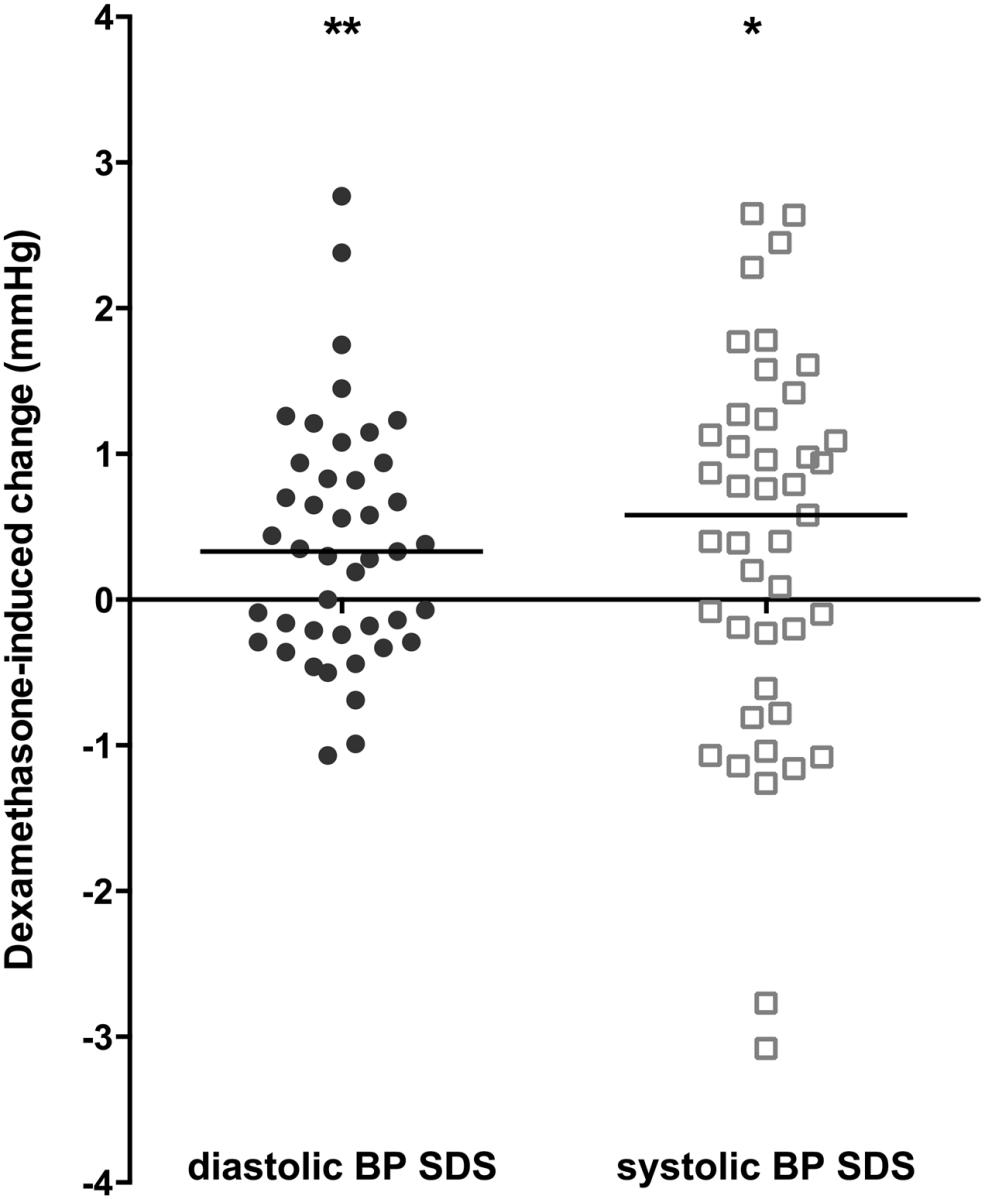
Blood pressure. Dexamethasone-induced changes in diastolic and systolic blood pressure (BP) SDS values after four days of dexamethasone treatment. N = 43. * = P<0.05 and ** = P<0.01 (Wilcoxon signed-rank test). The horizontal lines indicate the median change.

Blood pressure was measured for 43 patients. Dexamethasone treatment significantly increased both diastolic blood pressure SDS (median increase: 0.33, IQR: -0.24, 0.94; *P*<0.01) and systolic blood pressure SDS (median: 0.58, IQR: -0.61, 1.24; *P*<0.05). (Fig 6) The increase in diastolic blood pressure was inversely corelated with age (r = -0.43, *P*<0.01).

At baseline, 25% of patients had either diastolic or systolic hypertension (defined as a blood pressure value >95^th^ percentile, corrected for age and sex); this percentage increased to 39% after four days of dexamethasone treatment. Although this increase was not statistically significant (*P* = 0.14), it was inversely correlated with age (r = -0.36, *P* = 0.02).

### Components of metabolic syndrome

The International Diabetes Federation defines MetS in children >10 years of age as abdominal obesity, plus at least two of the following clinical features: hyperglycemia, hypertriglyceridemia, reduced HDL cholesterol, and hypertension. After four days of dexamethasone treatment, seven patients (three males) had abdominal obesity; the median age of these seven children was 9.0 years (IQR: 3.0, 12.0). Three of these seven patients developed hyperglycemia, hypertension, and/or hypertriglyceridemia (all of which are components of MetS) during the four days of dexamethasone treatment (Table 2). The oldest of these seven patients was 12 years of age and had a fasting glucose value of ≥5.6 mmol/L and high systolic blood pressure at T2. The other six patients were under 10 years of age and developed high blood pressure and hypertriglyceridemia following four days of dexamethasone treatment.

**Table 2:** Components of the metabolic syndrome.

	Abdominal obesity	Hypergly-cemia	Hypertriglyce-ridemia	Reduced HDL cholesterol	Hypertension	Abdominal obesity plus ≥2 MetS components
**T1** N (%)	5 (10)	0 (0)	10 (24)	2 (5)	11 (25)	0 (0)
**T2** N (%)	7 (15)	2 (5)	17 (41)	0 (0)	17 (39)	3 (7)

Incidence of clinical features of metabolic syndrome (MetS) at baseline (T1) and after four days of dexamethasone treatment (T2).

### Pharmacokinetics

The dexamethasone trough levels after four days of treatment were available for 24 patients; the median value was 3.97 μg/L (IQR: 1.45–11.81). Dexamethasone trough levels were not correlated with age, sex, or the week of maintenance phase. In addition, dexamethasone trough levels did not differ significantly between patients with abdominal obesity and patients without abdominal obesity (*P* = 0.17).

**Fig 7:**
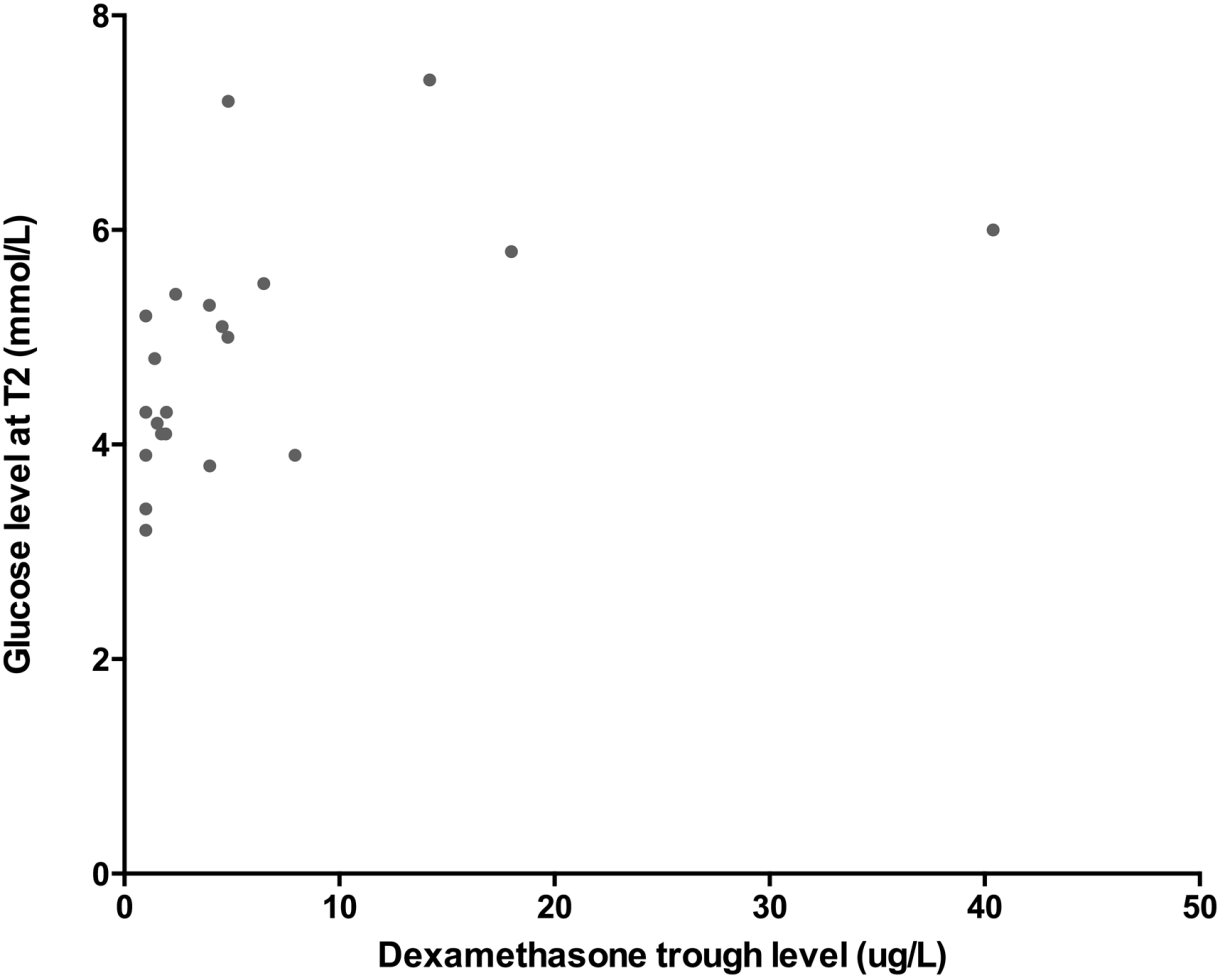
Dexamethasone levels and glucose. Glucose levels measured at T2 plotted against dexamethasone trough serum levels for each patient (N = 24). The two values were significantly correlated (Spearman’s coefficient (r): 0.63, P<0.01).

Dexamethasone trough levels were positively correlated with both the increase in glucose levels during dexamethasone treatment (r = 0.66, *P*<0.01) and glucose levels after four days of dexamethasone treatment (r = 0.63, *P*<0.01) (Fig 7). In contrast, dexamethasone trough levels were not correlated with insulin resistance, lipid serum levels, or blood pressures.

## Discussion

Here, we report the results of the first study to comprehensively investigate the acute effects of four days of dexamethasone treatment on all metabolic syndrome components in a relatively large cohort of 50 pediatric ALL patients. We found that one course of dexamethasone significantly increased insulin resistance and blood pressure, as well as fasting glucose and lipid levels, thereby satisfying three of the six factors associated with MetS (hyperglycemia, hypertriglyceridemia, and hypertension). Chow *et al* [6] reported that the prevalence of insulin resistance increased by 35.5% (N = 31 patients) during a single course of steroid (prednisone/dexamethasone) treatment; in contrast, the prevalence of insulin resistance increased by 67% in our cohort. This difference may be explained in part by the higher potency of 6 mg/m^2^ dexamethasone compared to 40 mg/m^2^ prednisolone. [1] The number of prior dexamethasone courses did not influence the metabolic effects of dexamethasone.

The acute effects of dexamethasone on metabolic function can have serious clinical consequences. For example, high glucose levels and insulin resistance can cause microvascular problems [35], impairmed function of the innate immune system [36], and changes in hemostatis. [37] Although the short-term effects of dexamethasone on metabolic function were not age-dependent, our results emphasize that even though MetS criteria are currently lacking for children under 10 years of age, young children with ALL are clearly susceptible to effects of dexamethasone treatment.

Over the long run, accumulating metabolic toxicity due to dexamethasone treatment during the one and a half years of maintenance therapy may contribute to the high incidence of MetS among survivors of pediatric leukemia [14, 38], thereby increasing their long-term risk for developing diabetes and/or cardiovascular disease. [14] Esbenshade *et al*. previously reported a 49% increase in insulin resistance during one year of maintenance therapy containing glucocorticoids [17], which supports our hypothesis of accumulating metabolic toxicity. By comparison, poor glucose control in pediatric patients with type 1 diabetes mellitus can increase long term complications. [39]

Dexamethasone-induced weight gain -which can be influenced by changes in eating behavior [3]—may contribute to the increased risk of long-term cardiovascular and diabetic complications. [3, 17] The prevalence of adiposity (determined based on patient BMI) in our population increased from 7% at the time of diagnosis to 19% at baseline (T1). This baseline prevalence of adiposity was higher in our cohort than in the Dutch general population (13%) [40], although the incidence of obesity (4%) was similar between our cohort and the Dutch population [40]. Moreover, abdominal adiposity and abdominal obesity -both of which are commonly reported steroid-related side effects [41]—were significantly more prevalent in our population than in the Dutch population. Thus, measuring waist circumference may have important clinical value, as steroids can cause a redistribution of fat from the extremities to the upper trunk and face. [41]

Another, dexamethasone-induced metabolic side effect is hypertension, which was reported previously in 45% of ALL patients during 28 days of prednisone-based induction therapy; 27% of these hypertensive patients received anti-hypertensive therapy. [7] We observed hypertension in 6% of our pediatric ALL patients after four days of dexamethasone treatment, and no anti-hypertensive drugs were administered. Dexamethasone-induced hypertension can contribute to inotropic and/or vasoconstrictive effects in the cardiovascular system. [41] steroid-induced hypertension.

A limitation of our study is that our patients also received vincristine, 6-mercaptopurine, and methotrexate during their course of dexamethasone treatment. Vincristine can induce the inappropriate secretion of anti-diuretic hormone, and 6-mercaptopurine and methotrexate can affect bone density; however, no other metabolic side effects have been linked to these drugs in pediatric ALL patients. [42] On the other hand, methotrexate has been linked to increases in LDL and HDL in adults with rheumatoid arthritis [43], which may explain the increase in HDL levels in our pediatric ALL patients and may reflect an influence of methotrexate on lipid levels.

Our analysis showed that dexamethasone trough levels were positively correlated with higher glucose levels after four days of dexamethasone treatment. These increased glucose levels could have been caused by an inhibition of glucose uptake in peripheral tissues and/or the stimulation of hepatic gluconeogenesis as a direct consequence of hepatic gene expression. [41] Our a priori hypothesis was that more metabolic parameters would be associated with dexamethasone clearance, as toxicity was previously associated with high dexamethasone levels. [44] Our finding that dexamethasone levels had no effect on lipids or insulin may have been the result of insufficient statistical power and/or the short-term dexamethasone exposure. Moreover, the pathophysiological mechanism of metabolic side effects—while not completely understood- may be side effect-specific [41, 45], which may explain the differences observed between metabolic side effects. These results underscore the need for large pharmacokinetics studies with long-term follow-up periods in order to investigate further the putative association between dexamethasone serum levels and metabolic toxicity. Given that dexamethasone trough levels varied widely among the patients in our cohort, achieving individualized dexamethasone dosing regimens is an important next step for treating children with ALL.

In conclusion, our results show that short-term dexamethasone treatment causes metabolic toxicity. Strikingly, only four consecutive days dexamethasone treatment significantly affected several key components of MetS in our pediatric ALL patients, and dexamethasone levels influenced glucose metabolism. These findings have direct clinical relevance, as they may form the basis for further studies regarding personalized treatment programs, including individualized dexamethasone dosing, targeted therapy with selective glucocorticoids [46], and programs designed to promote healthy diet and physical activity. This is an important point, as the accumulating of dexamethasone-induced metabolic toxicity -coupled with weight gain during dexamethasone treatment- may contribute to the increased prevalence of MetS and cardiovascular risk among survivors of childhood leukemia.

## Supporting Information

S1 File Study Protocol. (DOC)

S2 File CONSORT Checklist. (DOC)
