# Supplementary material for: Acute Activation of Metabolic Syndrome Components in Pediatric Acute Lymphoblastic Leukemia Patients Treated with Dexamethasone
Source: PLoS One. 2016 Jun 30;11(6):e0158225. doi: 10.1371/journal.pone.0158225 (PMC4928792; doi:10.1371/journal.pone.0158225)
Supplement: S1 File — (DOC) [file pone.0158225.s001.doc]

### PROTOCOL TITLE:

### Double blind randomized intervention study aiming at reducing dexamethasone related side effects in children with acute lymphoblastic leukemia (ALL).

| **Protocol ID** | **Dexadagenstudie/ 37826** |
| --- | --- |
| **Short title** | **Dexadagen studie** |
| **Version 3** |  |
| **Date** | **August 29th 2014** |
| **Coordinating investigator/project leader** | ***E.L.T. van den Akker, MD, PhD***  ***Pediatric endocrinologist***  ***Erasmus MC, Dr Molewaterplein 60***  ***3015 GJ Rotterdam***  [***e.l.t.vandenakker@erasmusmc.nl***](mailto:e.l.t.vandenakker@erasmusmc.nl)  ***Tel: +316 20596104*** |
| **Principal investigator(s) (in Dutch: hoofdonderzoeker/uitvoerder)**  ***Multicenter research: per site*** | ***Erasmus MC:***  ***E.L.T. van den Akker***  ***Pediatric endocrinologist***  ***M.M. van den Heuvel-Eibrink, MD, PhD***  ***Pediatric oncologist/ hematologist***  ***AMC:***  ***C. van den Bos, MD, PhD***  ***Pediatric oncologist/ hematologist***  ***UMCU;***  ***M.B. Bierings***  ***Pediatric oncologist/ hematologist***  ***Princess Máxima Center for Pediatric Oncology (PMC):***  ***R. Pieters, MD, PhD***  ***Pediatric oncologist/ hematologist***  ***UMCG:***  ***W.J.E. Tissing, MD, PhD***  ***Pediatric oncologist/ hematologist***  ***VUMC:***  ***M.A. Veening, MD, PhD*** |
|  | ***Pediatric oncologist/ hematologist*** |
| **Sponsor (in Dutch: verrichter/opdrachtgever)** | ***Erasmus MC***  ***Dr Molewaterplein 60***  ***3015 GJ Rotterdam*** |
|  |  |
| **Independent physician(s)** | ***Dr H.A.W.M. Tiddens (Erasmus MC)*** |
|  | ***Dr. A.M. Westermann (AMC)*** |
|  | ***J. Frenkel (UMCU)***  ***Dr. J.J. Boelens (PMC)*** |
|  | ***Dr. A.A. van de Loosdrecht (VUMC)*** |
|  | ***Dr. B.L. Rottier (UMCG)*** |
| **(Laboratory) sites <*if applicable*>** | ***M.L den Boer, PhD, Medical Biologist***  ***Laboratory Pediatric Oncology***  ***C.M. Zwaan, MD, PhD***  ***Dept of Pediatric oncology Erasmus MC*** |
| **Data Center and Monitoring** | ***Dutch Childhood Oncology Group - Early***  ***Clinical Trial Center (DCOG-ECTC)*** |
|  |  |
| **Pharmacy <*if applicable*>** | ***R. Zaal, pharmacologist***  ***Trial Pharmacy***  ***Erasmus MC*** |
|  |  |

Dutch Title:

Een dubbelblinde placebo gecontroleerde gerandomiseerde studie naar het verminderen van dexamethason gerelateerde bijwerkingen bij kinderen met leukemie (ALL).

Verkorte titel: ”Hoe overleef ik mijn dexadagen studie”.

**PROTOCOL SIGNATURE SHEET**

| **Name** | **Signature** | **Date** |
| --- | --- | --- |
| **Sponsor or legal representative:**  ***Erasmus MC***  ***Dr Molewaterplein 60***  ***3015 GJ Rotterdam***  **For non-commercial research,**  **Head of Department:**  ***Prof. dr. E.H.H.M. Rings***  ***Head Dept of Pediatrics***  ***Erasmus MC*** |  |  |
| **Coordinating Investigator/Project leader/Principal Investigator:**  ***E.L.T. van den Akker / M.M. van den Heuvel-Eibrink*** |  |  |
|  |  |  |

**TABLE OF CONTENTS**

1. INTRODUCTION and RATIONALE

: [1](#__RefHeading___Toc317680986)0

2. OBJECTIVES [14](#__RefHeading___Toc317681000)

3. STUDY DESIGN [14](#__RefHeading___Toc317681001)

4. STUDY POPULATION [15](#__RefHeading___Toc317681003)

5. TREATMENT OF SUBJECTS [15](#__RefHeading___Toc317681019)

6. INVESTIGATIONAL MEDICINAL PRODUCT [16](#__RefHeading___Toc317681022)

*7.* METHODS [17](#__RefHeading___Toc317681025)

7.1 Study parameters/endpoints [17](#__RefHeading___Toc317681026)

7.2 Randomisation, blinding and treatment allocation [20](#__RefHeading___Toc317681039)

7.3 Study procedures [21](#__RefHeading___Toc317681040)

7.4 Withdrawal of individual subjects [21](#__RefHeading___Toc317681041)

7.4.1 Specific criteria for withdrawal (if applicable) [21](#__RefHeading___Toc317681042)

7.5 Replacement of individual subjects after withdrawal [21](#__RefHeading___Toc317681043)

7.6 Follow-up of subjects withdrawn from treatment [21](#__RefHeading___Toc317681044)

7.7 Premature termination of the study [21](#__RefHeading___Toc317681045)

8 SAFETY REPORTING [22](#__RefHeading___Toc317681046)

8.2 Section 10 WMO event [22](#__RefHeading___Toc317681047)

8.3 Adverse and serious adverse events [22](#__RefHeading___Toc317681048)

8.3.1 Suspected unexpected serious adverse reactions (SUSAR) [24](#__RefHeading___Toc317681049)

8.3.2 Annual safety report [25](#__RefHeading___Toc317681050)

8.4 Follow-up of adverse events [25](#__RefHeading___Toc317681051)

8.5 Data Safety Monitoring Board (DSMB) [25](#__RefHeading___Toc317681052)

9 STATISTICAL ANALYSIS [25](#__RefHeading___Toc317681053)

9.2 Descriptive statistics [26](#__RefHeading___Toc317681057)

9.3 Univariate analysis [26](#__RefHeading___Toc317681058)

9.4 Multivariate analysis [26](#__RefHeading___Toc317681059)

9.5 Interim analysis (if applicable) [27](#__RefHeading___Toc317681060)

10.1 Regulation statement [27](#__RefHeading___Toc317681061)

10.2 Recruitment and consent [27](#__RefHeading___Toc317681062)

10.3 Objection by minors or incapacitated subjects (if applicable) [27](#__RefHeading___Toc317681063)

10.4 Benefits and risks assessment, group relatedness [27](#__RefHeading___Toc317681064)

10.5 Compensation for injury [28](#__RefHeading___Toc317681065)

10.6 Incentives (if applicable) [28](#__RefHeading___Toc317681066)

11. ADMINISTRATIVE ASPECTS AND PUBLICATION [28](#__RefHeading___Toc317681067)

11.1 Handling and storage of data and documents [28](#__RefHeading___Toc317681068)

11.2 Amendments [29](#__RefHeading___Toc317681069)

11.3 Annual progress report [29](#__RefHeading___Toc317681070)

11.4 End of study report [29](#__RefHeading___Toc317681071)

11.5 Public disclosure and publication policy [30](#__RefHeading___Toc317681072)

12. MONITORING AND PATIENT SAFETY PLAN [30](#__RefHeading___Toc317681073)

13. EXPECTED OUTLINE OF MANUSCRIPTS……………………………………………..29

14. REFERENCES………………………………………………………………………………31

15. ADDENDUM: Questionnaire after parent or patient refusal……………………………37

**LIST OF ABBREVIATIONS AND RELEVANT DEFINITIONS**

| **ABR** | **ABR form, General Assessment and Registration form, is the application form that is required for submission to the accredited Ethics Committee (In Dutch, ABR = Algemene Beoordeling en Registratie)** |
| --- | --- |
| **AE** | **Adverse Event** |
| **AR** | **Adverse Reaction** |
| **CA** | **Competent Authority** |
| **CCMO** | **Central Committee on Research Involving Human Subjects; in Dutch: Centrale Commissie Mensgebonden Onderzoek** |
| **CV** | **Curriculum Vitae** |
| **DSMB** | **Data Safety Monitoring Board** |
| **EU** | **European Union** |
| **EudraCT** | **European drug regulatory affairs Clinical Trials** |
| **GCP** | **Good Clinical Practice** |
| **IB** | **Investigator’s Brochure** |
| **IC** | **Informed Consent** |
| **IMP** | **Investigational Medicinal Product** |
| **IMPD** | **Investigational Medicinal Product Dossier** |
| **METC** | **Medical research ethics committee (MREC); in Dutch: medisch ethische toetsing commissie (METC)** |
| **(S)AE** | **(Serious) Adverse Event** |
| **SPC** | **Summary of Product Characteristics (in Dutch: officiële productinfomatie IB1-tekst)** |
| **Sponsor** | **The sponsor is the party that commissions the organisation or performance of the research, for example a pharmaceutical**  **company, academic hospital, scientific organisation or investigator. A party that provides funding for a study but does not commission it is not regarded as the sponsor, but referred to as a subsidizing party.** |
| **SUSAR** | **Suspected Unexpected Serious Adverse Reaction** |
| **Wbp** | **Personal Data Protection Act (in Dutch: Wet Bescherming Persoonsgevens)** |
| **WMO** | **Medical Research Involving Human Subjects Act (in Dutch: Wet Medisch-wetenschappelijk Onderzoek met Mensen** |

**SUMMARY**

**Rationale:** The use of dexamethasone (a synthetic corticosteroid) is an essential component for effective treatment of childhood acute lymphoblastic leukemia (ALL). However, it has been reported that in 5-75% of children, treatment with dexamethasone, is accompanied by serious side-effects on mood, cognition and behaviour leading to major effects on wellbeing of children and their parents. In addition, these side-effects often induce therapy adjustments with the risk of decreased effect and subsequent outcome. The exact pathophysiology of the dexamethasone-induced cerebral side-effects is unknown but has been presumed to be mediated by its effect on the glucocorticoid receptor. Dexamethasone suppresses endogenous production of cortisol. Recent findings show that lack of endogenous cortisol can lead to such side-effects by lack of stimulation of the mineralocorticoid receptor (MR) in the brain. Cortisol dependent mineralocorticoid receptor effects in the brain have been shown to be involved in emotion, memory and sleep. These recent findings led to our novel hypothesis: During treatment with dexamethasone, the deprived mineralocorticoid receptor may cause serious cerebral side effects. Hence, it is feasible that these side effects on mood, behaviour and cognition could be prevented, by an intervention with a natural occurring hormone that stimulates the mineralocorticoid receptor in the brain in a physiological way. This can be done by adding physiological dosages of cortisol during dexamethasone treatment. To ensure the safety of this study, we performed a preclinical study, in which we found that adding cortisol to both in *vitro* cultured leukemic cell lines and *ex vivo* cultured primary patients’ cells, did not influence the anti-leukemic effect of dexamethasone. Previously, we and others have shown that *in vitro* dexamethasone sensitivity is strongly associated with clinical steroid response and with clinical outcome.

**Objective**:

- The primary aim of the study is to reduce dexamethasone induced cerebral side-effects on mood, behaviour, and cognition by adding physiological doses of cortisol to standard treatment
- The secondary aim of the study is to analyse the effect of the intervention on other side-effects of dexamethasone; a descriptive intra-patient comparison of blood pressure, insulin levels, visceral fat gain, lipid profile and glucose levels between intervention treatment and placebo.
- The third aim of the study is to determine the positive predictive value positive predictive value of the salivary very low dose dexamethasone suppression test with cortisol as a novel in vivo diagnostic test, on dexamethasone side-effects.

**Study design:** A prospective double blind placebo-controlled randomized cross-over design.

**Study population:** 50 patients, aged 3-16 years, treated according to medium risk ALL treatment schedule will be included after informed consent.

**Intervention**: During 2 identical periods of 5 days of dexamethasone treatment, in addition, patients will receive either a physiological dose of cortisol (intervention) or placebo.

**Main study parameters/endpoints:**

- Questionnaires: the parent-reported strength and difficulty questionnaire in Dutch (SDQ-Dut) ^20^ after 5 days of dexamethasone treatment with or without cortisol. This will be the primary outcome parameter. The other questionnaires will be secondary outcome parameters.
- Bloodtest results: cortisol, lipid spectrum etc
- Measurement of blood pressure, BMI, weight
- Complaints diary
- Food dairy
- Neuropsychological test
- Physical activity

**Nature and extent of the burden and risks associated with participation, benefit and group relatedness:** Extent and burden is low.

The risk-benefit analysis for the study shows a favorable risk profile. The investigational medicinal product (IMP) that is studied is hydrocortisone, given in a physiological dose, NOT in a pharmacological dose. No side effects are expected in this dose. In addition, we performed pre-clinical in vitro and ex vivo studies to prove that adding cortisol to treatment will not have any negative effects on the efficacy of cytotoxic effect on leukemia cells.

Participants will fill in 4 questionnaires on mood, behavior and daily activity, 1 dietary diary (24 hour) at home. They will have 4 neuropsychological tests on cognition, memory and attention during hospital visits (total extra time: 2 hours/visit). In addition they are asked to wear a accelerometer (3x3 cm on their belt) for 2x 5 days (intervention and placebo period) to measure physical activity. Before start of the intervention they will perform once a salivary diurnal cortisol rhythm and a diagnostic salivary very low dose (0,25 mg) dexamethasone suppression test at home, which involves taking 4 salivary samples one day, ingestion of 0,25 mg dexamethasone (weight adjusted) in the evening and one salivary sample the next day. Blood sampling will be done 4 times 3 ml, using the existing vascular access ports, during regular hospital visits, when routine blood tests are already planned.

The intervention drug is widely used and we have ample experience with this drug. The intervention drug is a hydrocortisone, given in a *physiological* dose, NOT in a pharmacological dose. No side effects are expected in this dose. In addition we performed pre-clinical in vitro and ex vivo studies to prove that adding cortisol to treatment will not have any negative effects on leukemia treatment. As the administered dose of cortisol in the intervention arm of the study is a physiological dose, we do not expect major risks, and hence we will not install a data and safety monitoring board (DSMB) for this study.

Although it is not primary aim of this study, participants can benefit from the effects of the intervention. The benefit is that this study has the potential to have major consequences for the quality of life for all childhood ALL patients.

**1. INTRODUCTION AND RATIONALE**

Leukemia is the most frequent cancer in children. A corner stone component of treatment is the use of corticosteroids of which dexamethasone seems the most effective as the anti-leukemic activity of dexamethasone is 7-16-fold higher than that of prednisolone^1^. Besides systemic effects, dexamethasone has also a good central nervous system penetration, thereby preventing CNS disease and is therefore used in many current treatment schedules^2-5^. Counterpart of the treatment with dexamethasone and prednisolone is the occurrence of serious side effects^6-7^ on metabolism (like diabetes mellitus, hypertension, hyperlipidemia) and on the brain.

Belgaumi et al compared the toxicity of dexamethasone and prednisone in ALL patients. The incidence of hypertension in 50 ALL patients treated with dexamethasone was 10%. For hyperglycemia the incidence was 20% in the same group. Dexamethasone led to more weight gain than did prednisone (11.9% vs.5.4%; P=0.002).^54^

Wallace et al found that glucocorticoid administration for five weeks in 19 ALL patients resulted in significant increased adiposity and insulin resistance. Dexamethasone was significantly more potent than prednisolone in altering these parameters.^55^

Bostrom et al reported higher incidence of hyperglycemia and grades 1 to 3 steroid myopathy in patients treated with dexamethasone compared to prednisone. ^56^

Children and their families often find the emotional and behavioral aberrations caused by the side-effects of dexamethasone to be the most deleterious to quality of life.^8-9^ In 5-75% of children, treatment with dexamethasone, is accompanied by serious side-effects on the brain like mood, cognition and behavioural problems, sometimes even resulting in psychosis or depression^8-12^. The impact of these side effects of dexamethasone is considerable and gives risk to discontinuation of treatment, at the cost of a higher risk of treatment failure.

Normally endogenous cortisol (figure 1a) binds to the glucocorticoid receptor and the mineralocorticoid receptor in the brain in a ratio of 1:1. Dexamethasone has a 30-40 fold higher potency to activate the glucocorticoid receptor than cortisol. Historically, it is presumed that the high occupation of the glucocorticoid receptor is causing the side effects. However, in dexamethasone treated patients, the mineralocorticoid receptor is not occupied because the production of cortisol, which binds the mineralocorticoid receptor in healthy persons, is suppressed by the negative feedback of dexamethasone on the hypothalamus-pituitary-adrenal axis(fig1b).^13^

Recent research data suggest that mineralocorticoid receptors in the brain play an important role in the regulation of complex behavior, such as emotion, cognition and sleep.^14^ In animal studies, mice treated with mineralocorticoid antagonists had impaired spatial memory and learning. Mineralocorticoid knockout mice show increased anxiety behavior^14^. In humans, treatment with mineralocorticoid antagonists has been associated with impaired selective attention, impaired recall of visuospatial memory and diminished slow wave sleep^14^. The differential effects of natural and synthetic corticosteroids on the brain caused by different binding properties to the glucocorticoid receptor and mineralocorticoid receptor are also illustrated by examples from different patient categories. Many Addison patients, who lack endogenous cortisol, experience complaints of mood and impairment of cognitive function. Addison patients were found to have their best performance on cognitive function tests when treated with dexamethasone *and* cortisol compared to dexamethasone only^15^. The clinical implications were also illustrated in a case report of a 48-year-old woman who needed synthetic corticosteroid treatment after bilateral adrenalectomy for Cushing’s syndrome. She developed severe psychotic symptoms that were unresponsive to psychotropic drugs as long as she was taking prednisolone as replacement therapy. Prednisolone has an effect on the glucocorticoid receptor and the mineralocorticoid receptor with a ratio of 5:1. However, after she was switched to a regimen of cortisol and fludrocortisone that lead to more occupation of the mineralocorticoid receptor, the psychopathology disappeared^16^.

These recent findings led to our new hypothesis (Fig 1c) that dexamethasone induced cortisol depletion of the mineralocorticoid receptor in the brain, causes the serious side effects on mood, behavior and sleep, which can be resolved by adding a physiologic dose of cortisol to treatment (Figure 3). The hypothesis had also not been tested in adult leukemia patients treated with dexamethasone.

Before this hypothesis could be tested in patients, we performed a pre-clinical in vitro study to prove that adding cortisol to treatment will not have any negative effects on leukemia treatment.

Dexamethasone induced cell apoptosis of leukemia cells has been shown to be dependent on dexamethasone binding to the cortisol receptor.^17^

In the preclinical pilot study, responsiveness of leukemia cells to cortisol and dexamethasone was determined by the methyl-thiazol-tetrazolium (MTT) salt drug cytotoxicity assay (MTT-assay) (Fig 2, see addendum). This assay has been optimized to *ex vivo* test sensitivity of leukemic cells of patients^18^, which den Boer JCO 2003 is predictive for the long-term clinical outcome of pediatric ALL patients^19^. Firstly, glucocorticoid resistant and sensitive leukemic cell lines were studied. Secondly, *ex vivo* patient leukemia cells were used to study dexamethasone and prednisolone induced cell kill with and without adding cortisol (Fig 2, see addendum). We found that adding cortisol to both in *vitro* cultured leukemic cell lines and *ex vivo* cultured primary patients’ cells, did not influence in vitro dexamethasone nor prednisolone sensitivity of ALL cells.

# 2. OBJECTIVES

Primary aim

- To reduce dexamethasone induced cerebral side-effects on mood, behaviour, and cognition by intervention treatment with physiological doses of cortisol compared to placebo.

Secondary aims

- To study other dexamethasone toxicities; a descriptive intra-patient comparison of blood pressure, insulin levels, visceral fat gain, lipid profile and glucose levels between intervention treatment and placebo.
- To study the positive predictive value of novel in vivo diagnostic tests (consisting of salivary very low dose dexamethasone suppression test), on dexamethasone side-effects

# STUDY DESIGN

A double blind placebo-controlled randomized cross-over design.

This study will be a multicenter study. Study coordination and randomization will be performed within the Dutch Childhood Oncology Early Clinical Trial Center localized in Erasmus MC/Sophia.

The study protocol will be performed in children treated according to the Dutch DCOG ALL-10 or 11 protocols, or the relapse ALL R3 protocol, which are National DCOG protocols.

*.*

# STUDY POPULATION

**Patients**: After informed consent, ALL patients, aged 3-16 years, treated according to the DCOG upfront ALL10 (or ALL11) - MRG or ALL R3 relapse protocol in maintenance phase are eligible for the study. The study will be performed during a period where neither anthracyclines nor asparaginase are used. The age range is 3-16 years as the primary outcome parameters (questionnaires) are validated for this age range. As ALL patients are currently already treated according to these protocols, we expect to be able to start enrollment of patients shortly after regulatory approvals (METC and CCMO) and institutional permissions are obtained.

**Inclusion criteria:**

- written informed consent

- age 3-16

- histologically or cytologically confirmed acute lymphoblastic leukemia

- inclusion in DCOG ALL10 or ALL11 protocol or ALL R3 protocol

- able to comply with scheduled follow-up.

**Exclusion criteria**:

- patient or parent refusal

- anticipated compliance problems

- underlying conditions which affect the absorption of oral medication

- pregnant or lactating patients

- current uncontrolled infection or any other complication which may interfere with dexamethasone treatment

- language barrier

- preexisting mental retardation syndrome

# TREATMENT OF SUBJECTS

In this double-blind placebo-controlled cross-over study patients receive the IMP or the placebo during 2 periods of a 5 day dexamethasone treatment. All children will be randomized into two groups: either dexa + IMP or dexa + placebo first. After a washout period of 2 weeks and 2 days, a new medication period is started where the group that had a placebo in the first period will receive the study drug and the other group will get the placebo.

## Investigational medicinal product/treatment

The intervention product is cortisol suspension, given orally. This drug is given in a physiological dose of 10 mg/m2/day. Patients will use the hydrocortisone solution (1mg/ml) or placebo 3 times daily orally (5:3:2 ratio / circadian rhythm). Timing of intake: first dose after awakening. Second dose between 12am and 1pm, the third dose will be between 6pm and 8 pm. Study medication will be taken at the same time as the dexamethasone. The pharmacy will prepare the IMP as well as a placebo suspension. Further information on the IMP is being prepared by the pharmacy.

Schedule for the two study Dexamethasone courses

| **Time** | **Day 1** | **Day 2** | **Day 3** | **Day 4** | **Day 5** |
| --- | --- | --- | --- | --- | --- |
| **8-10am** | Dexa + study med/placebo | Dexa + study med/placebo | Dexa + study med/placebo | Dexa + study med/placebo | Dexa + study med/placebo |
| **12am-2pm** | Dexa + study med/placebo | Dexa + study med/placebo | Dexa + study med/placebo | Dexa + study med/placebo | Dexa + study med/placebo |
| **6-8pm** | Dexa + study med/placebo | Dexa + study med/placebo | Dexa + study med/placebo | Dexa + study med/placebo | Dexa + study med/placebo |

Table 1*. Dexamethasone 6 mg/m2/day oral, divided in 3 doses . Study medication: hydrocortison (1mg/ml) in a physiological dose of 10 mg/m2/day divided in 3 doses with a circadian rhythm( ratio 5:3:2). Placebo will be given in same dose.*

**Use of co-intervention (if applicable)**

Not applicable

## Escape medication (if applicable)

Not applicable

# INVESTIGATIONAL MEDICINAL PRODUCT

## The investigational medicinal product in this study will be: hydrocortisone. All relevant details are described and available in the IMPD as separate reference document. A seraparate IMPD of the placebo is available.

.

## Drug accountability

We will control intake of medication (placebo and hydrocortisone) by measuring of the fluid volume in the medicine bottles after each 5 day course of study medication. The bottles will be destroyed after use by the pharmacy. Fluid measurement will be done in the oncology department. We will also control dexamethasone intake which will be recorded in the patient diary.

All used, dispensed, and destroyed medication will be recorded and monitored.

# *7.* METHODS

## 7.1 Study parameters/endpoints

**Intervention**: We will study 50 children in a cross-over design. During dexamethasone treatment, patients will receive either placebo or cortisol in a physiological dose of 10 mg/m2/day divided in 3 doses with a circadian rhythm. Cortisol is available and registered for pediatric use in several oral preparations. No side-effects are expected because it is used in physiological dosages and long term experience with this drug has been obtained in various other patient groups.

Primary **outcome parameter** is the parent-reported strength and difficulty questionnaire in Dutch (SDQ-Dut) ^20^ after 5 days of dexamethasone treatment with or without cortisol. In the general population mean SDQ score is 7,0 (1 SD= 6,0)^20^ . We expect the mean SDQ-Dut score of the children after 5 days of dexamethasone treatment to be around 15 in the control group. Our aim is to have a SDQ score in the treated group, which is 1 SD lower (6 points) compared to the controls. Secondary outcome parameters are mood and behaviour questionnaires and (neuro-) cognitive tests and metabolic parameters (see below).

*Fig 2. Schematic study design*

**ALL M-risk**

**Informed consent**

**Salivary suppr. test**

**Questionnaires (4 )**

**Neurocognitive tests**

**Metabolic parameters (5ml blood)**

**Dietary diary (24hr), accelerometer**

**Dexamethasone +**

**Hydrocortisone (3x/day)**

**Dexamethasone**

**+ placebo (3x/day)**

**Dexamethasone +**

**hydrocortisone (3x/day)**

**Dexamethasone**

**+ placebo (3x/day)**

**5 days Dexa course**

**5 days Dexa course**

***Fig.2 :*** *Medium risk ALL patients will be randomised after informed consent in to two groups. One week before start of the dexamethasone course they will perform a salivary dexamethasone suppression test.*

*(Neuro-) psychological outcome parameters*

Dexamethasone induced side-effects on mood, behavior and cognition will be analyzed before and on day 5 of dexamethasone treatment with a set of validated neuropsychological assessment:

Neuropsychological tests and questionnaires developed for children and their parents were administered to assess skills in six domains in 30 minutes:

1. Behavior/mood: Strength and Difficulties Questionnaire in Dutch (SDQ-Dut)^20^ = primary outcome parameter.
2. Eating behavior: Dutch Eating Behavior Questionnaire (NVE-K)^21^
3. Sleeping disorders: Dutch version of Children’s sleep habits questionnaire^22^
4. Visual-spatial functions: Design copying (NEPSY II)^23^
5. Memory: Narrative memory and Memory for design (NEPSY II)^23^
6. Attention: Auditory Attention and Response Set, and Inhibition (NEPSY II)^23^
7. Speed: Substitution and Symbol Search (aged appropriate Wechsler; WPSSI II or WISC III)^24-25^.
8. Dietary diary (24 hours)
9. Daily activity: Baecke questionnaire
10. Physical activity (5 day measurement) with Philips device
11. Diary of complaints: (during to study dexamethasone courses) also contains times of medication intake

*Metabolic outcome parameters*

Data on body composition will be obtained. Height and weight will be measured. Waist-hip circumpherence and blood pressure will be measured. Fasting blood samples will be taken to measure lipid profiles (triglycerides, cholesterol, HDL, LDL), glucose and insulin levels allowing calculation of the homeostatic model assessment (HOMA)-index for evaluation of insulin sensitivity levels, lipid profiles. Some serum will be stored. The outcome parameters will be analysed separately for children above the age of six, the diagnosis of metabolic syndrome will be assessed based on IDF (international diabetes federation) criteria^26^.This international consensus definition is a simple and easy-to-apply clinical definition facilitating international comparison.

*Individual corticosteroid sensitivity parameters*

For individual profiles of corticosteroid sensitivity the following data will be obtained: salivary diurnal cortisol rhythm (area under the curve), salivary very low dose (0,25 mg) dexamethasone suppression test^27^. This will be performed one week before start of the first study dexamethasone course and can be done at home. Salivary samples could be send to the endocrinology labarotory.

Table 2.: **Schedule Dexamethasone suppression test at home**

| **Time** | **Day 1** | **Day 2** |
| --- | --- | --- |
| awakening | Salivary sample (sober) | Salivary sample (sober) |
| 12am | Salivary sample |  |
| 4 pm | Salivary sample |  |
| 8 pm | Salivary sample |  |
| 8pm | 0.25mg dexa oral |  |

*Visits outpatient clinic*

Neuropsychological and metabolic outcome parameters will be obtained in both study courses at the first day and the fifth day of the dexamethasone course. At these days the patients will have to complete the questionnaires, fasting blood samples will be taken and neuropsychological test will be administered. Patients usually have a routine visit at the outpatient clinic the first day of each dexamethasone course. The fifth day study visit will be an extra visit for each patient.

# Randomisation, blinding and treatment allocation

Subjects will be recruited from a population of children and adolescents treated at or referred to the investigational centers.

After informed consent is obtained and after eligibility screening the site will enroll

subjects into the study. Patients with informed consent but who fail to meet the eligibility criteria are screen failures.

Patient registration procedure will be available via the website <http://www.skion.nl/dcog-ectc/studies>. This method of registration will be done utilizing a web-based service called TenALEA which will be available 24 hours 7 days per week. Notification of a registration will be send automatically to all relevant parties via email. A subject number will be assigned at this time. The subject number will be recorded in the patient medical file.

The pharmacy department of the Erasmus MC will upon receipt of the registration notification randomise the subject using a computerized program. The randomisation result will be notified to the requesting parties in a blinded manner. The result will be recorded in the patient medical file.

In case any enrolment issues need to be discussed prior to electronic randomisation, please send an e-mail to both PIs simultaneously for discussion:

e.l.t.vandenakker@erasmusmc.nl and m.vandenheuvel@erasmusmc.nl, and send a copy to research-kocr@erasmusmc.nl

It is the responsibility of the Investigator to ensure that the subject is eligible for the study before enrolling the subject.

To register the subject via TenALEA the following information will be required:

1. Gender, Date of Birth

2. Leukemia Type and Phase; Date of Diagnosis

3. Review of Eligibility Criteria

4. Expected Date of Study Treatment Start.

Blinding will be continued till the ‘last patient-last visit’ (LPLV).

## Study procedures

See above, section 7.1

## Withdrawal of individual subjects

Subjects can leave the study at any time for any reason if they wish to do so without any consequences. The investigator can decide to withdraw a subject from the study for urgent medical reasons. In case of grade III/IV clinically relevant toxicities, which can’t be managed by standard procedures and interfere with the neuropsychological tests at day 5 of the dexamethasone courses, the investigator will decide to postpone study medication. The subject can restart study medication in the following dexamethasone course.

### Specific criteria for withdrawal (if applicable)

## Replacement of individual subjects after withdrawal

Enrolled subjects are all subjects who signed the informed consent form.

Evaluable subjects: All subjects who received two courses of study treatment (one placebo and one trial medication) and two on-treatment efficacy evaluations. Analyses of efficacy will be performed on the dataset of all efficacy-evaluable subjects.

Subjects will be enrolled until a total of 50 evaluable subjects is reached.

Patients who aren’t able to get dexamethasone or who have to quit during a course will have

to start over with study medication at a new following course of dexamethasone.

## Follow-up of subjects withdrawn from treatment

Treated subjects are all subjects who received at least one dose of study treatment. Demographic and baseline characteristics and safety analyses will be performed on all treated subjects.

## Premature termination of the study

The Principal Investigators can decide to premature termination of the study on the following criteria:

- There is evidence of an unacceptable risk for trial subjects (i.e. safety issue)

- There is reason to conclude that it will not be possible to collect the data necessary to

reach the study objectives and it is therefore not ethical to continue enrolment of more

patients. The PI’s will notify the METC and the competent authority within 15 days, including the reasons for the premature termination.

# SAFETY REPORTING

## Section 10 WMO event

In accordance to section 10, subsection 1, of the WMO, the investigator will inform the subjects and the reviewing accredited METC if anything occurs, on the basis of which it appears that the disadvantages of participation may be significantly greater than was foreseen in the research proposal. The study will be suspended pending further review by the accredited METC, except insofar as suspension would jeopardise the subjects’ health. The investigator will take care that all subjects are kept informed.

## Adverse and serious adverse events

Adverse events are defined as any undesirable experience occurring to a subject during the study, whether or not considered related to [the investigational product / the experimental treatment]. All adverse events reported spontaneously by the subject or observed by the investiga­tor or his staff will be recorded.

A serious adverse event is any untoward medical occurrence or effect that at any dose:

- results in death;
- is life threatening (at the time of the event);
- requires hospitalisation or prolongation of existing inpatients’ hospitalisation;
- results in persistent or significant disability or incapacity;
- is a congenital anomaly or birth defect;
- is a new event of the trial likely to affect the safety of the subjects, such as an unexpected outcome of an adverse reaction, lack of efficacy of an IMP used for the treatment of a life threatening disease, major safety finding from a newly completed animal study, etc.

**NOTE** 1:

• Pregnancy: Incidence of pregnancy is not considered a SAE; pregnancy must, however, be reported immediately by E-mail to the principal investigators of this study;

• Overdose: All cases of overdose must be reported immediately by E-mail to the principal investigators of this study.

**NOTE 2**:

Criteria for hospitalizations not reported as SAEs include admissions for:

- Planned as per protocol medical/surgical procedure

- Routine health assessment requiring admission for baseline/trending of health status documentation

- Medical/surgical admission for purpose other than remedying ill health state (planned prior to entry into study trial; appropriate documentation required)

- Admission encountered for other life circumstance that carries no bearing on health status and requires no medical/surgical intervention (e.g. lack of housing, economic inadequacy, care-giver respite, family circumstances, administrative)

- Admissions for protocol-scheduled procedures or blood product transfusions will not be considered SAEs.

All SAEs occurring during the study or 16 days after the last administration of the trial medication, must be reported to the principal investigator within 24 hours of occurrence in order to comply with regulatory requirements. Adverse events classified as "serious" must be recorded on the SERIOUS AE (SAE) page of the CRF.

SAE reporting by TELEPHONE, FAX and E-MAIL:

DCOG –ECTC Safety Desk, Tel 010-70 36325 / 36402, Fax 010-703 6681

E-mail to: [research-kocr@erasmusmc.nl](mailto:research-kocr@erasmusmc.nl), [e.l.t.vandenakker@erasmusmc.nl](mailto:e.l.t.vandenakker@erasmusmc.nl), [m.vandenheuvel@erasmusmc.nl](mailto:m.vandenheuvel@erasmusmc.nl) and [l.warris@erasmusmc.nl](mailto:l.warris@erasmusmc.nl)

Collection of complete information concerning SAEs is extremely important. If only limited information is initially available, follow-up reports are required. Thus, follow-up information which becomes available as the SAE evolves, as well as supporting documentation (e.g., hospital discharge summaries and autopsy reports), should be collected subsequently, if not available at the time of the initial report, and immediately sent using the same procedure as the initial SAE report. For ongoing SAEs a follow-up report should be sent at least once-monthly. The investigator is responsible for submitting these follow-up reports for all SAEs, until the SAE has resolved or until the patient’s condition stabilizes (in the case of persistent impairment), or the patient dies.

All SAEs will be reported through the web portal *ToetsingOnline* to the accredited METC that approved the protocol, within 15 days after the sponsor has first knowledge of the serious adverse reactions.

SAEs that result in death or are life threatening should be reported expedited. The expedited reporting will occur not later than 7 days after the responsible investigator has first knowledge of the adverse reaction. This is for a preliminary report with another 8 days for completion of the report.

### Suspected unexpected serious adverse reactions (SUSAR)

Adverse reactions are all untoward and unintended responses to an investigational product related to any dose administered.
Unexpected adverse reactions are adverse reactions, of which the nature, or severity, is not consistent with the applicable product information (e.g. Investigator’s Brochure for an unapproved IMP or Summary of Product Characteristics (SPC) for an authorised medicinal product).
The principal investigators are responsible for reporting SAEs/SUSARS to the IRB or other applicable regulatory authority.

In accordance with local regulations, the trial bureau will notify Investigators of all AEs that are serious, unexpected, and certainly, probably, or possibly related to the investigational product. This notification will be in the form of a SUSAR report.

Upon receiving such notices, the Investigator must review and retain the SUSAR reports with the Investigator Brochure. Where required by local regulations or when there is a central Institutional Review Board (IRB)/Independent Ethics Committee (IEC) for the study, the Sponsor will submit the SUSAR report to the appropriate IRB/IEC. The sponsor, together with the principal investigators, will determine if the informed consent requires revision.

The sponsor will report expedited the following SUSARs through the web portal *ToetsingOnline* to the METC:

- SUSARs that have arisen in the clinical trial that was assessed by the METC;

The remaining SUSARs are recorded in an overview list (line-listing) that will be submitted once every half year to the METC. This line-listing provides an overview of all SUSARs from the study medicine, accompanied by a brief report highlighting the main points of concern.

The expedited reporting of SUSARs through the web portal ToetsingOnline is sufficient as notification to the competent authority.

The sponsor will report expedited all SUSARs to the competent authorities in other Member States, according to the requirements of the Member States.

The expedited reporting will occur not later than 15 days after the sponsor has first knowledge of the adverse reactions. For fatal or life threatening cases the term will be maximal 7 days for a preliminary report with another 8 days for completion of the report.

### Annual safety report

In addition to the expedited reporting of SUSARs, the sponsor will submit, once a year throughout the clinical trial, a safety report to the accredited METC, competent authority, Medicine Evaluation Board and competent authorities of the concerned Member States.

This safety report consists of:

- a list of all suspected (unexpected or expected) serious adverse reactions, along with an aggregated summary table of all reported serious adverse reactions, ordered by organ system, per study;
- a report concerning the safety of the subjects, consisting of a complete safety analysis and an evaluation of the balance between the efficacy and the harmfulness of the medicine under investigation.

## Follow-up of adverse events

All adverse events will be followed until they have abated, or until a stable situation has been reached. Depending on the event, follow up may require additional tests or medical procedures as indicated, and/or referral to the general physician or a medical specialist.

## Data Safety Monitoring Board (DSMB)

A DSMB will not be installed in this study. The patients are treated for a short

time frame (5 days) and the drugs under investigation are well characterised and are given in a physiological dose, which is known for not harming patients. However, the data will be monitored as described in section 12 of this protocol.

# STATISTICAL ANALYSIS

The particular strength of this cross over design is that the interventions under investigation are evaluated within the same patients and so eliminates between-subject variability.

To prevent carry-over effect of treatments across study periods, a wash-out period is present and with the blinded randomisation the patient will start with either placebo or trial medication in a randomised fashion.

Given that the subjects act as their own controls, the analyses are based on paired data analysis.

Primary outcome parameter is the SDQ (continuous parameters). The effectiveness of treatments is assessed by comparing the effects of placebo and trial medication on SDQ by a two-way analysis of variance, with treatment order as a between-subjects factor and treatment type as a within-subject factor.

Secondary outcome parameters are mood and behaviour questionnaires, (neuro-) cognitive tests and metabolic parameters (all continuous parameters). The effectiveness of treatments is assessed by comparing the effects of placebo and trial medication on secondary outcome parameters by a two-way analysis of variance, with treatment order as a between-subjects factor and treatment type as a within-subject factor.

## The end of treatment point is at the end of day 5 of the second dexamethasone study course after completion of the neuropsychological tests. There is no follow-up planned or required except for monitoring of serious adverse events.

## Sample size calculation

Power analysis was performed on the mean outcome parameter SDQ. The mean of the SDQ scale is expected to be 7,0 with a standard deviation of 6,0. In the placebo effect we expect a rise of 8 points and a correlation of 64% between measurement at start and finish of the medication period. This correlation will result in a larger power as we will perform an analysis with the second measurement as outcome and first measurement and intervention as predictors.

Iizuka et al found that children with ADHD, who have behavioural and concentration problems, have a mean SDQ score of 16, which is one standard deviation above the general population.^57^ After simulating five hundred datasets we found that 40 children is enough to have 80% chance of finding a significant relationship (alpha = 0.05) when the effect of the intervention is 6 points (1 SD) on the scale. Considering the probability of drop-out, a total of 50 patients will be included.

## Descriptive statistics

Descriptive statistics (N, arithmetic mean, standard deviation, minimum, median, maximum, percent coefficient of variation, and geometric mean) are presented. Missing data will be omitted from the calculation of descriptive statistics. Summaries about the sample and the measures are presented in tables and / or graphs.

## Univariate analysis

See above

## Multivariate analysis

See above

## Interim analysis (if applicable)

*Not applicable*

# ETHICAL CONSIDERATIONS

## Regulation statement

The study will be performed according to the declaration of Helsinki (09.10.2004)

and the Medical Research Involving Human Subjects Act (WMO) and the Good

Clinical Practice standard.

## Recruitment and consent

Patients will be asked to participate in the study by their pediatric oncologist and the patient information letter and informed consent form are provided. Patients will have at least 2 weeks to consider their decision, or longer if more time is needed. If patients decide not to participate in the study after being informed about the study, they will be offered to complete a voluntary questionnaire about the reason they are not participating and the type of side effects of dexamethasone they experience. This questionnaire is completely voluntary, so patients are not obliged to give a reason for not participating in the study. We will use these questionnaires to gain more information about the severity of dexamethasone side effects in the non-participating patients.

## Objection by minors or incapacitated subjects (if applicable)

The code for minors is applicable for this project (ccmo.nl).

Pediatric acute lymphoblastic leukemia is a disease in children in the age category of birth - 18 years. The study aims at reducing side effects in the treatment of this disease. Therefore, children are included in the study. In children < 12 years of age, informed consent is given by the parents/ caregivers. In children of 12 years and older, informed consent is given by the child and the parents/caregivers.

## Benefits and risks assessment, group relatedness

Given the very low risk and low to moderate patient burden of this intervention study, and that this study has the potential to have major consequences for the improvement of quality of life for all childhood ALL patients , this study is considered to have a favorable risk-benefit assessment

.

## Compensation for injury

The sponsor/investigator has a liability insurance which is in accordance with article 7, subsection 6 of the WMO.

The sponsor (also) has an insurance which is in accordance with the legal requirements in the Netherlands (Article 7 WMO and the Measure regarding Compulsory Insurance for Clinical Research in Humans of 23th June 2003). This insurance provides cover for damage to research subjects through injury or death caused by the study.

1. € 450.000,-- (i.e. four hundred and fifty thousand Euro) for death or injury for each subject who participates in the Research;
2. € 3.500.000,-- (i.e. three million five hundred thousand Euro) for death or injury for all subjects who participate in the Research;
3. € 5.000.000,-- (i.e. five million Euro) for the total damage incurred by the organisation for all damage disclosed by scientific research for the Sponsor as ‘verrichter’ in the meaning of said Act in each year of insurance coverage.

The insurance applies to the damage that becomes apparent during the study or within 4 years after the end of the study.

## Incentives (if applicable)

Not applicable

# ADMINISTRATIVE ASPECTS AND PUBLICATION

## Handling and storage of data and documents

This study will be conducted in accordance with the ethical principles that have their origin in the current Declaration of Helsinki, and will be consistent with International Conference on Harmonization Good Clinical Practice (ICH GCP) and applicable regulatory requirements.

The study will be conducted in compliance with the protocol. The protocol and any Amendments and the subject informed consent will receive Institutional Review Board (IRB)/Independent Ethics Committee (IEC) approval/favorable opinion prior to initiation of the study.

The rights, safety and well-being of the trial subjects are the most important considerations and should prevail over interests of science and society.

Study personnel involved in conducting this trial will be qualified by education, training, and experience to perform their respective task(s), and will be authorized to perform study related procedures as laid down in the delegation of authority log.

Systems with procedures that assure the quality of every aspect of the study will be implemented.

## Amendments

A ‘substantial amendment’ is defined as an amendment to the terms of the METC application, or to the protocol or any other supporting documentation, that is likely to affect to a significant degree:

- the safety or physical or mental integrity of the subjects of the trial;
- the scientific value of the trial;
- the conduct or management of the trial; or
- the quality or safety of any intervention used in the trial.

All substantial amendments will be notified to the METC and to the competent authority.

Non-substantial amendments will not be notified to the accredited METC and the competent authority, but will be recorded and filed by the sponsor.

## Annual progress report

The sponsor/investigator will submit a summary of the progress of the trial to the accredited METC once a year. Information will be provided on the date of inclusion of the first subject, numbers of subjects included and numbers of subjects that have completed the trial, serious adverse events/ serious adverse reactions, other problems, and amendments.

## End of study report

The sponsor will notify the accredited METC and the competent authority of the end of the study within a period of 90 days. The end of the study is defined as the last patient’s last visit.

In case the study is ended prematurely, the sponsor will notify the accredited METC and the competent authority within 15 days, including the reasons for the premature termination.

Within one year after the end of the study, the investigator/sponsor will submit a final study report with the results of the study, including any publications/abstracts of the study, to the accredited METC and the Competent Authority.

## Public disclosure and publication policy

This trial is registered in the Dutch trial registry. The results of this study will be disclosed unreservedly.

# MONITORING AND PATIENT SAFETY PLAN

The drugs under investigation are well characterised and are given in a physiological dose, which is known for not harming patients. Therefore we consider a minimal risk for adverse effects. We have to upgrade our risk classification to medium risk because our study group consists of vulnerable children. The monitoring frequency on site will be 2-3 times a year. Monitoring will be performed by an independent monitor of the DCOG -ECTC. Monitoring extend and other details will be described in the Monitor Manual.

# Expected outline of manuscripts:

1. In vitro and ex vivo dexamethasone sensitivity of acute lymfoblastic leukemia cells is not influenced by adding cortisol. Auteurs: Warris, van den Heuvel-Eibrink, Pieters, van den Akker, den Boer.

2. Dexamethasone induced cerebral side-effects on mood, behaviour and cognition in children with Acute Lymphoblastic Leukemia. A Cochrane review. Warris, van den Heuvel-Eibrink, Pieters, Aarsen, van den Akker.

3. The effect of addition of cortisol to dexamethasone treatment on cognition in children with ALL. A double blind randomised trial. Warris, van den Akker, Zwaan, van den Bos, Bierings, Tissing, Veening, Aarsen, Pieters, van den Heuvel-Eibrink

4. The effect of combining cortisol and dexamethasone on mood and behaviour in children with ALL. A double blind randomised trial. Warris, van den Heuvel-Eibrink, Zwaan, van den Bos, Bierings, Tissing, Veening, Pieters, van den Akker
 
5. The risk of metabolic syndrome during ALL treatment with (cortisol and) dexamethasone. Warris, van den Akker, Zwaan, van den Bos, Bierings, Tissing, Veening, Rotteveel, Pieters, van den Heuvel-Eibrink

6. The positive predictive value of the very low dose dexamethasone suppression test, a novel in vivo diagnostic test, on severity of dexamethasone side-effects. Warris, van den Heuvel-Eibrink, van den Akker

***14.* REFERENCES**

1. Kaspers GJ, Veerman AJ, Popp-Snijders C, Lomecky M, Van Zantwijk CH, Swinkels LM,

Van Wering ER, Pieters R. Comparison of the antileukemic activity in vitro of dexamethasone and prednisolone in childhood acute lymphoblastic leukemia. Med Pediatr Oncol 1996;27:114-21.

2. Kamps WA, Veerman AJ, van Wering ER, van Weerden JF, Slater R, van der Does-van den Berg A. Long-term follow-up of Dutch Childhood Leukemia Study Group (DCLSG) protocols for children with acute lymphoblastic leukemia, 1984-1991. Leukemia 2000;14:2240-6.

3. Veerman AJ, Kamps WA, van den Berg H, van den Berg E, Bokkerink JP, Bruin MC, van

den Heuvel-Eibrink MM, Korbijn CM, Korthof ET, van der Pal K, Stijnen T, van Weel Sipman MH, van Weerden JF, van Wering ER, van der Does-van den Berg A, Dutch Childhood Oncology G. Dexamethasone-based therapy for childhood acute lymphoblastic leukaemia: results of the prospective Dutch Childhood Oncology Group (DCOG) protocol ALL-9 (1997-2004). Lancet Oncol 2009;10:957-66.

4. Balis FM, Lester CM, Chrousos GP, Heideman RL, Poplack DG. Differences in cerebrospinal fluid penetration of corticosteroids: possible relationship to the prevention of meningeal leukemia. J Clin Oncol 1987;5:202-7.

5. Veerman AJ, Hahlen K, Kamps WA, Van Leeuwen EF, De Vaan GA, Solbu G, Suciu S, Van Wering ER, Van der Does-Van der Berg A. High cure rate with a moderately intensive treatment regimen in non-high-risk childhood acute lymphoblastic leukemia. Results of protocol ALL VI from the Dutch Childhood Leukemia Study Group. J Clin Oncol 1996;14:911-8.

6. te Winkel ML, Appel IM, Pieters R, van den Heuvel-Eibrink MM. Impaired dexamethasone-related increase of anticoagulants is associated with the development of osteonecrosis in childhood acute lymphoblastic leukemia. Haematologica 2008;93:1570-4.

7. van der Sluis IM, van den Heuvel-Eibrink MM. Osteoporosis in children with cancer.

Pediatr Blood Cancer 2008;50:474-8; discussion 86.

8. McGrath P, Pitcher L. 'Enough is enough': qualitative findings on the impact of dexamethasone during reinduction/consolidation for paediatric acute lymphoblastic leukaemia. Support Care Cancer 2002;10:146-55.

9. Hochhauser CJ, Lewis M, Kamen BA, Cole PD. Steroid-induced alterations of mood and

behavior in children during treatment for acute lymphoblastic leukemia. Support Care Cancer

2005;13:967-74.

10. Satel SL. Mental status changes in children receiving glucocorticoids. Review of the

literature. Clin Pediatr (Phila) 1990;29:383-8.

11. Stuart FA, Segal TY, Keady S. Adverse psychological effects of corticosteroids in children and adolescents. Arch Dis Child 2005;90:500-6.

12. Brown ES, Suppes T. Mood symptoms during corticosteroid therapy: a review. Harv Rev

Psychiatry 1998;5:239-46.

13. Lustig RH. Corticosteroid replacement therapy. In: Lin AN, Paget SA, eds. Principles of

corticosteroid therapy. New York: Arnold; 2002:205-20.

14. Kellner M, Wiedemann K. Mineralocorticoid receptors in brain, in health and disease:

possibilities for new pharmacotherapy. Eur J Pharmacol 2008;583:372-8.

15. Tytherleigh MY, Vedhara K, Lightman SL. Mineralocorticoid and glucocorticoid receptors

and their differential effects on memory performance in people with Addison's disease.

Psychoneuroendocrinology 2004;29:712-23.

16. Seifritz E, Hemmeter U, Poldinger W, Froesch ER, Reul JM, Holsboer-Trachsler E.

Differential mood response to natural and synthetic corticosteroids after bilateral adrenalectomy: a case report. J Psychiatr Res 1994;28:7-11.

17. Helmberg A, Auphan N, Caelles C, Karin M. Glucocorticoid-induced apoptosis of human

leukemic cells is caused by the repressive function of the glucocorticoid receptor. Embo J

1995;14:452-60.

18. Pieters R, Huismans DR, Loonen AH, Hahlen K, van der Does-van den Berg A, van Wering ER, Veerman AJ. Relation of cellular drug resistance to long-term clinical outcome in childhood acute lymphoblastic leukaemia. Lancet 1991;338:399-403.

19. Kaspers GJ, Pieters R, Van Zantwijk CH, Van Wering ER, Van Der Does-Van Den Berg A, Veerman AJ. Prednisolone resistance in childhood acute lymphoblastic leukemia: vitro-vivo correlations and cross-resistance to other drugs. Blood 1998;92:259-66.

20. van Widenfelt BM, Goedhart AW, Treffers PD, Goodman R. Dutch version of the Strengths and Difficulties Questionnaire (SDQ). Eur Child Adolesc Psychiatry 2003;12:281-9.

14

21. van Strien T. Nederlandse Vragenlijst voor Eetgedrag bij kinderen. Amsterdam; 2007.

22. van Litsenburg RR, Waumans RC, van den Berg G, Gemke RJ. Sleep habits and sleep

disturbances in Dutch children: a population-based study. Eur J Pediatr 2010;169:1009-15.

23. Kirk U, Korkman M, kemp S. NEPSY II. Amsterdam; 2010.

24. Hendriksen J, Hurks P. Wechsler Preschool and Primary Scale of Intelligence. Amsterdam; 2009.

25. Kort W, Schittekatte M, Bosmans M, Compaan E, Dekker PH, Vermeir G, Verhaeghe P.

Wechsler Intelligence Scale for children III. Amsterdam; 2005.

26. Zimmet P, Alberti G, Kaufman F, Tajima N, Silink M, Arslanian S, Wong G, Bennett P,

Shaw J, Caprio S, International Diabetes Federation Task Force on E, Prevention of D. The

metabolic syndrome in children and adolescents. Lancet 2007;369:2059-61.

27. Huizenga NA, Koper JW, de Lange P, Pols HA, Stolk RP, Grobbee DE, de Jong FH, Lamberts SW. Interperson variability but intraperson stability of baseline plasma cortisol concentrations, and its relation to feedback sensitivity of the hypothalamo-pituitary-adrenal axis to a low dose of dexamethasone in elderly individuals. J Clin Endocrinol Metab 1998;83:47-54.

28. Russcher H, Smit P, van Rossum EF, van den Akker EL, Brinkmann AO, de Heide LJ, de

Jong FH, Koper JW, Lamberts SW. Strategies for the characterization of disorders in cortisol

sensitivity. J Clin Endocrinol Metab 2006;91:694-701.

29. van den Akker EL, Koper JW, Joosten K, de Jong FH, Hazelzet JA, Lamberts SW, Hokken-Koelega AC. Glucocorticoid receptor mRNA levels are selectively decreased in neutrophils of children with sepsis. Intensive Care Med 2009;35:1247-54.

30. van den Akker EL, Koper JW, Joosten K, de Jong FH, Hazelzet JA, Lamberts SW, Hokken- Koelega ACS. Glucocorticoid receptor expression is decreased in neutrophils of children with sepsis. Int Care Med 2009;35:1247-54.

31. van den Akker EL, Koper JW, van Rossum EF, Dekker MJ, Russcher H, de Jong FH,

Uitterlinden AG, Hofman A, Pols HA, Witteman JC, Lamberts SW. Glucocorticoid receptor gene and risk of cardiovascular disease. Arch Intern Med 2008;168:33-9.

32. van den Akker EL, Nouwen JL, Melles DC, van Rossum EF, Koper JW, Uitterlinden AG,

Hofman A, Verbrugh HA, Pols HA, Lamberts SW, van Belkum A. Staphylococcus aureus nasal carriage is associated with glucocorticoid receptor gene polymorphisms. J Infect Dis

2006;194:814-8.

33. van den Akker EL, Russcher H, van Rossum EF, Brinkmann AO, de Jong FH, Hokken A,

Pols HA, Koper JW, Lamberts SW. Glucocorticoid receptor polymorphism affects transrepression but not transactivation. J Clin Endocrinol Metab 2006;91:2800-3.

34. Voorhoeve PG, van den Akker EL, Lamberts SW, Delemarre-van de Waal HA, Hokken-

Koelega AC. Glucocorticoid receptor gene polymorphism is less frequent in children born small for gestational age without catch-up growth. Horm Res 2009;71:162-6.

35. van Beek RD, Bezemer DD, Meijerink JP, de Muinck Keizer-Schrama SM, Haas OA, Te

Winkel L, Pieters R, van den Heuvel-Eibrink M. Repeats in the kringle IV encoding domains in the Apo(a) gene and serum lipoprotein(a) level do not contribute to the risk for avascular necrosis of the bone (AVN) in pediatric acute lymphoblastic leukemia. Leukemia 2006;20:879-80.

36. van Beek RD, Smit M, van den Heuvel-Eibrink MM, de Jong FH, Hakvoort-Cammel FG,

van den Bos C, van den Berg H, Weber RF, Pieters R, de Muinck Keizer-Schrama SM. Inhibin B is superior to FSH as a serum marker for spermatogenesis in men treated for Hodgkin's lymphoma with chemotherapy during childhood. Hum Reprod 2007;22:3215-22.

37. van Beek RD, van den Heuvel-Eibrink MM, Hakvoort-Cammel FG, van den Bos C, van

der Pal HJ, Krenning EP, de Rijke YB, Pieters R, de Muinck Keizer-Schrama SM. Bone mineral density, growth, and thyroid function in long-term survivors of pediatric Hodgkin's lymphoma treated with chemotherapy only. J Clin Endocrinol Metab 2009;94:1904-9.

38. van Beek RD, van den Heuvel-Eibrink MM, Laven JS, de Jong FH, Themmen AP,

Hakvoort-Cammel FG, van den Bos C, van den Berg H, Pieters R, de Muinck Keizer-Schrama SM. Anti-Mullerian hormone is a sensitive serum marker for gonadal function in women treated for Hodgkin's lymphoma during childhood. J Clin Endocrinol Metab 2007;92:3869-74.

39. van Beek RD, de Muinck Keizer-Schrama SM, Hakvoort-Cammel FG, van der Sluis IM,

Krenning EP, Pieters R, van den Heuvel-Eibrink MM. No difference between prednisolone and dexamethasone treatment in bone mineral density and growth in long term survivors of childhood acute lymphoblastic leukemia. Pediatr Blood Cancer 2006;46:88-93.

40. Lie Fong S, Laven JS, Hakvoort-Cammel FG, Schipper I, Visser JA, Themmen AP, de Jong FH, van den Heuvel-Eibrink MM. Assessment of ovarian reserve in adult childhood cancer survivors using anti-Mullerian hormone. Hum Reprod 2009;24:982-90.

41. Hartman A, te Winkel ML, van Beek RD, de Muinck Keizer-Schrama SM, Kemper HC, Hop WC, van den Heuvel-Eibrink MM, Pieters R. A randomized trial investigating an exercise

program to prevent reduction of bone mineral density and impairment of motor performance

during treatment for childhood acute lymphoblastic leukemia. Pediatr Blood Cancer 2009;53:64-71.

42. Buizer AI, De Sonneville LM, van den Heuvel-Eibrink MM, Njiokiktjien C, Veerman AJ.

Visuomotor control in survivors of childhood acute lymphoblastic leukemia treated with

chemotherapy only. J Int Neuropsychol Soc 2005;11:554-65.

43. Buizer AI, de Sonneville LM, van den Heuvel-Eibrink MM, Veerman AJ. Chemotherapy

and attentional dysfunction in survivors of childhood acute lymphoblastic leukemia: effect of

treatment intensity. Pediatr Blood Cancer 2005;45:281-90.

44. Buizer AI, de Sonneville LM, van den Heuvel-Eibrink MM, Veerman AJ. Behavioral and

educational limitations after chemotherapy for childhood acute lymphoblastic leukemia or Wilms tumor. Cancer 2006;106:2067-75.

45. Pieters R, Kaspers GJ, Klumper E, Veerman AJ. Clinical relevance of in vitro drug

resistance testing in childhood acute lymphoblastic leukemia: the state of the art. Med Pediatr Oncol 1994;22:299-308.

46. van den Heuvel-Eibrink MM, Wiemer EA, de Boevere MJ, Slater RM, Smit EM, van

Noesel MM, van der Holt B, Schoester M, Pieters R, Sonneveld P. MDR1 expression in poor-risk acute myeloid leukemia with partial or complete monosomy 7. Leukemia 2001;15:398-405.

47. van den Heuvel-Eibrink MM, Wiemer EA, de Boevere MJ, van der Holt B, Vossebeld PJ,

Pieters R, Sonneveld P. MDR1 gene-related clonal selection and P-glycoprotein function and

expression in relapsed or refractory acute myeloid leukemia. Blood 2001;97:3605-11.

48. Boot AM, van den Heuvel-Eibrink MM, Hahlen K, Krenning EP, de Muinck Keizer-

Schrama SM. Bone mineral density in children with acute lymphoblastic leukaemia. Eur J Cancer 1999;35:1693-7.

49. Tissing WJ, Meijerink JP, Brinkhof B, Broekhuis MJ, Menezes RX, den Boer ML, Pieters R. Glucocorticoid-induced glucocorticoid-receptor expression and promoter usage is not linked to glucocorticoid resistance in childhood ALL. Blood 2006;108:1045-9.

50. Tissing WJ, Meijerink JP, den Boer ML, Pieters R. Molecular determinants of glucocorticoid sensitivity and resistance in acute lymphoblastic leukemia. Leukemia 2003;17:17-25.

51. van Casteren NJ, Dohle GR, Romijn JC, de Muinck Keizer-Schrama SM, Weber RF, van

den Heuvel-Eibrink MM. Semen cryopreservation in pubertal boys before gonadotoxic treatment and the role of endocrinologic evaluation in predicting sperm yield. Fertil Steril 2008;90:1119-25.

52.Bruni O, Ottaviano S, Romoli M, Innocenzi M, Cortesi F, Giannotti F. The sleep disturbance scale for children (SDSC). Construction and validation of an instrument to evaluate sleep disturbances in childhood and adolescence. Journal of sleep research. 1996; 5:251-261: Dutch translation WTP Verheij & RJEM Raymann 2005.

53. Vogels N, Westerterp KR, Posthumus DLA, Rutters F, Westerterp-Plantenga MS. Daily

physical activity counts vs structured activity counts in lean and overweight Dutch children.

Physiology and Behavior 2007; 92: 611-616.

54. Belgaumi AF, Al-Bakrah M, Al-Mahr M, Al-Jefri A, Al-Musa A, Saleh M, Salim MF, Osman M, Osman L, El-Solh H. Dexamethasone-Associated Toxicity during Induction Chemotherapy for Childhood Acute Lymphoblastic Leukemia Is Augmented by Concurrent Use of Daunomycin. Cancer 2003; 97:2898–903.

55. Wallace AM, Tucker P, Williams DM, Hughes IA, Ahmed SF. [Short-term effects of prednisolone and dexamethasone on circulating concentrations of leptin and sex hormone-binding globulin in children being treated for acute lymphoblastic leukaemia.](http://www.ncbi.nlm.nih.gov/pubmed/12780755) [Clin Endocrinol (Oxf)](http://www.ncbi.nlm.nih.gov/pubmed#%23) 2003; 58(6):770-6.

56. Bostrom BC, Sensel MR, Sather HN, Gaynon PS, La MK, Johnston K, Erdmann GR, Gold S, Heerema NA, Hutchinson RJ, Provisor AJ, Trigg ME; Children’s Cancer Group. Dexamethasone versus prednisone and daily oral versus weekly intravenous mercaptopurine for patients with standard-risk acute lymphoblastic leukemia: a report from the Children's Cancer Group. Blood, 2003; 101(10):3809-17.

57. [Iizuka C](http://www.ncbi.nlm.nih.gov/pubmed?term="Iizuka C"%5BAuthor%5D), [Yamashita Y](http://www.ncbi.nlm.nih.gov/pubmed?term="Yamashita Y"%5BAuthor%5D), [Nagamitsu S](http://www.ncbi.nlm.nih.gov/pubmed?term="Nagamitsu S"%5BAuthor%5D), [Yamashita T](http://www.ncbi.nlm.nih.gov/pubmed?term="Yamashita T"%5BAuthor%5D), [Araki Y](http://www.ncbi.nlm.nih.gov/pubmed?term="Araki Y"%5BAuthor%5D), [Ohya T](http://www.ncbi.nlm.nih.gov/pubmed?term="Ohya T"%5BAuthor%5D), [Hara M](http://www.ncbi.nlm.nih.gov/pubmed?term="Hara M"%5BAuthor%5D), [Shibuya I](http://www.ncbi.nlm.nih.gov/pubmed?term="Shibuya I"%5BAuthor%5D), [Kakuma T](http://www.ncbi.nlm.nih.gov/pubmed?term="Kakuma T"%5BAuthor%5D), [Matsuishi T](http://www.ncbi.nlm.nih.gov/pubmed?term="Matsuishi T"%5BAuthor%5D). Comparison of the strengths and difficulties questionnaire (SDQ) scores between children with high-functioning autism spectrum disorder (HFASD) and attention-deficit/hyperactivity disorder (AD/HD). Brain Dev., 2010; 32(8):609-12.

15. Formulier afzien van deelname

Titel van het onderzoek: **”Hoe overleef ik mijn dexadagen”**

(Officiële titel: Double blind randomized intervention study aiming at reducing dexamethasone related side effects in children with acute lymphoblastic leukemia (ALL)

Ik heb besloten om NIET aan het onderzoek, of een deel daarvan, deel te nemen.

Om een indruk te krijgen van de mate van klachten van dexamethason onder de kinderen die niet deelnemen aan het onderzoek, zouden we graag een korte vragenlijst af nemen.

Deze vragenlijst is geheel vrijwillig. Het is dus **niet verplicht** onderstaand gedeelte in te vullen.

Ik wil wel / niet* via dit formulier de reden aangeven, waarom ik niet deelneem aan het onderzoek.

Zo ja, dan kunt u verder gaan met de volgende vraag.

Mijn reden voor afzien van deelname is:

ik heb geen interesse

ik heb al aan zoveel onderzoeken meegedaan

ik heb geen klachten van dexamethason

ik heb er geen tijd voor

anders, nl. …………………………………………..……………………………………………………..

…………………………………………..……………………………………………………..

Om toch een globaal inzicht te geven in een aantal zaken die van belang zijn voor het onderzoek ben ik wel bereid een 10-tal vragen te beantwoorden (dit is niet verplicht):

nee

ja, zie vragen hieronder

1. Heeft uw zoon/dochter klachten (gehad) van de dexamethason blokken op het gebied van stemming, gedrag en denken?

nee, sla vraag 2 t/m7 over en vervolg met vraag 8

ja

1. Is/was er sprake van stemmingsklachten?

ja

nee

1. Is/was er sprake van agressie?

ja

nee

1. Is/was er sprake van ongeremde eetbuien?

ja

nee

1. Is/was er sprake van slaapproblemen?

ja

nee

1. Is/was er sprake van concentratieproblemen?

ja

nee

1. Is/was er sprake van geheugenproblemen?

ja

nee

1. Is/was er sprake van gewichtstoename tijdens de dexamethason blokken?

ja

nee

1. Is/was er sprake van schommelingen van het suikergehalte (hypoglykemie /hyperglykemie) tijdens de dexamethason blokken?

ja, namelijk ……………………………………………………………………….

nee

1. Moest uw zoon/dochter insuline gebruiken tijdens de dexamethason blokken?

ja

nee

Ondertekening:

Naam: ………………………………..………………………………

Datum: ……………………….. Handtekening: ………………………..……………

| Dexadagen studie  versie 5-06-2012 |  |  |
| --- | --- | --- |

* doorhalen wat niet van toepassing is.
